# Heterogeneous distribution of *k13* mutations in *Plasmodium falciparum* in Laos

**DOI:** 10.1186/s12936-018-2625-6

**Published:** 2018-12-27

**Authors:** Moritoshi Iwagami, Masami Nakatsu, Phonepadith Khattignavong, Pheovaly Soundala, Sengdeuane Keomalaphet, Lavy Lorpachan, Phonepadith Xangsayalath, Emilie Matsumoto-Takahashi, Virginie Pommelet, Bouasy Hongvanthong, Paul T. Brey, Shigeyuki Kano

**Affiliations:** 1 Department of Tropical Medicine and Malaria, Research Institute, National Centre for Global Health and Medicine, 1-21-1 Toyama, Shinjuku, Tokyo 162-8655 Japan; 2 SATREPS Project (JICA/AMED) for Parasitic Diseases, Vientiane, Lao People’s Democratic Republic; 3 Institut Pasteur du Laos, Ministry of Health, Sansenthai Road, Ban Kao-Gnot, Sisattanak District, P.O. Box 3560, Vientiane, Lao People’s Democratic Republic; 4 National Institute of Public Health, Ministry of Health, Vientiane, Lao People’s Democratic Republic; 5 Center of Malariology, Parasitology and Entomology, Ministry of Health, Vientiane, Lao People’s Democratic Republic

**Keywords:** Artemisinin-resistance, Laos, *k13*, Malaria, *Plasmodium falciparum*, Greater Mekong Sub-region

## Abstract

**Background:**

The emergence and transnational spread of artemisinin resistance in *Plasmodium falciparum* in the Greater Mekong Sub-region (GMS) is a serious threat to malaria elimination in the region and could present a threat to malaria control in Africa. Recently, the Lao Government adopted the goal of malaria elimination by 2030, for which monitoring of artemisinin-resistant malaria within the country is indispensable. This study’s objectives were to assess the distribution of *k13* mutations in Laos.

**Methods:**

*Plasmodium falciparum* isolates (n = 1151) were collected from five southern provinces in Laos between 2015 and 2016, and three isolates from the northernmost province bordering China in 2017. Polymorphisms of the *k13* gene and two flanking regions were analysed to estimate relationship among the isolates.

**Results:**

In the five southern provinces, overall 55.5% of the isolates possessed artemisinin-resistant mutations of the *k13* gene (C580Y, P574L, R539T, Y493H). The C580Y was the predominant mutation (87.2%). The frequencies of the *k13* mutations were heterogeneous in the five southern provinces, but with a clear tendency showing the highest frequency in the south (72.5%) and to a lower degree when moving northward (28.0%). The three isolates from the Lao–Chinese border also possessed the C580Y mutation. Analysis of the flanking loci demonstrated that these three isolates were genetically very close to resistant strains originating from western Cambodia.

**Conclusions:**

Artemisinin resistance was observed to be rapidly increasing and spreading northwards through Laos and has now reached the Chinese border. The Lao and Chinese governments, as well as the international community, should make dedicated efforts to contain the spread of *k13* mutations within Laos and in the GMS.

**Electronic supplementary material:**

The online version of this article (10.1186/s12936-018-2625-6) contains supplementary material, which is available to authorized users.

## Background

Extensive efforts have reduced the burden of malaria in the Greater Mekong Sub-region (GMS) including Laos, also known as the Lao People’s Democratic Republic (Lao PDR) [1]. Recently, the Lao government adopted the goal of eliminating malaria by 2030 [2]. However, there are many challenges to overcome to achieve this goal. First, in Laos, artemisinin-based combination therapy (ACT) is the first-line treatment regimen for both *Plasmodium falciparum* and *Plasmodium vivax* [1]; however, therapeutic efficacy studies conducted in the southern part of Laos in 2013 demonstrated that 2.2% (Salavan province), 22.2% (Champasak province) [3] and 5.2% (Attapeu province) [4] of the patients treated with artemether–lumefantrine, one ACT, were still parasitaemic on day 3 after treatment. Second, mutations in a gene-encoding kelch-propeller domains in chromosome 13 in *P. falciparum* (*k13*) are widely used as primary determinant of artemisinin-resistance [5, 6], and this genetic marker enables the monitoring of the spread of artemisinin-resistance [7–10]. The previous study showed an average of 20.0% (24/122) of *P. falciparum* isolates collected in the southern provinces of Laos in 2013 (Savannakhet Champasak, Salavan) possessed artemisinin-resistant mutations in the *k13* gene [11]. In another study, as much as 77.5% (86/111) of the isolates collected in Champasak in 2014 possessed the C580Y mutation in the *k13* gene, which is one of the predominant artemisinin-resistant mutations in the GMS [12]. The rapid increase and distribution of the artemisinin-resistant *k13* mutations in Laos is of major concern.

Blood samples were collected from October 2015 to April 2016 together with socio-demographic and clinical data from malaria patients who visited public healthcare facilities in the five southern provinces (Savannakhet, Salavan, Sekong, Attapeu, Champasak) where 96.5% of malaria cases in Laos were reported in 2014 [13]. Blood samples were also collected from malaria-suspected patients in Phongsaly, the northernmost province, which is adjacent to China. In addition, the associations between the *k13* polymorphisms and demographic and clinical profiles were estimated. This survey provides the latest status on increase and spread of the *k13* polymorphisms in the southern and northern parts of Laos, which is crucial for making and updating drug policy to accelerate the elimination of falciparum malaria in the country, as well as safeguarding against the further spread of resistant malaria beyond the country’s borders.

## Methods

### Study design, sampling and oversight

Investigators from National Centre for Global Health and Medicine (NCGM), Japan and Institut Pasteur du Laos (IPL) designed the study based on malaria epidemiological data in 2013 summarized by the Centre of Malariology, Parasitology and Entomology, Lao Ministry of Health. This study has three components: training of local healthcare facilities staff, malaria patient blood sampling and data collection, and DNA analysis. Two-five malaria high endemic districts were selected in each province based on annual parasite incidence in 2013. A total of 163 public healthcare facilities (hospitals, health centres and malaria sections in Provincial and District Health Offices in the selected districts) were chosen for this study (Additional file 1). Finger-prick blood samples were collected from malaria patients (microscopic or rapid diagnostic test (RDT) positive) on filter paper (Whatman™ FTA™ Classic Cards, GE Healthcare Life Science, UK) [13, 14] from the five southern provinces from October 2015 through April 2016. Finger-prick blood samples were also collected from malaria suspected patients on filter paper from Phongsaly, the northernmost province, from November to December 2017. Those suspected malaria patients consisted of both positive and negative patients by microscopy or RDT. Socio-demographic and clinical data were also obtained using questionnaire forms. The blood samples were sent to the NCGM for DNA analysis after obtaining authorization from the Lao Ministry of Health.

The research proposal was reviewed and approved by the National Ethics Committee for Health Research, Ministry of Health, Lao PDR (No. 049 NIOPH/NECHR) in 2014. Written informed consent was obtained from all the participants prior to interview and the collection of blood for the diagnosis of malaria. The guardians of child participants (< 18 years old) consented to their participation.

### Genotyping

DNA extraction and PCR for *Plasmodium* species identification are shown in Additional file 2. Mutations in the *k13* gene were examined by the *k13* toolbox previously described [11]. When the *k13* gene region was not amplified by the primer sets in the toolbox, additional primer sets were designed and used (Additional file 3). When the mutation was observed in the *k13* gene in the sample, the PCR amplification and DNA sequencing were performed once again as an internal quality assessment. The sequencing results were analysed by ClustalW using the 3D7 kelch13 sequence as a reference (Accession: XM_001359122.1). The samples with mixed alleles (wild type and mutated type) were considered to be mutated isolates to estimate the frequency of the *k13* mutations. When two mutations were observed in the *k13* gene from a single patient, the PCR genotyping of two other gene regions (*msp1* and *msp2*) encoding merozoite surface proteins (MSP1 and MSP2) was performed [15], which allowed to differentiate “double mutations in the *k13* gene” from “multiple clone infection”.

Two flanking loci of the *k13* gene, PF3D7_1337500 (K13_151) and PF3D7_1339700 (K13_159), were amplified and DNA sequences were analysed (Additional file 3) [11]. Haplotypes were generated based on the combinations of the two loci to assess dissemination of the *k13* mutations (Additional files 4, 5, 6).

### Statistical analysis

Data were analysed with Microsoft Excel. For bivariate analyses, the Chi square test and Fisher’s exact test were used to evaluate an association between variables and *Plasmodium* infection. *P*-values less than 0.05 were considered statistically significant. Multivariate logistic regression analyses adjusted for the effects of other variables were conducted to estimate the association between variables and the *k13* mutated *Plasmodium* infection using SPSS version 18.0 (SPSS INC., Chicago, IL, USA) and STATA version 11 (StataCorp, USA). Several models were constructed using significant variables (*P* < 0.05) in the bivariate analyses.

## Results

A total of 2409 dried blood samples was collected on filter papers from malaria patients who were diagnosed by microscopy at hospitals or malaria RDT at health centres in the five southern provinces from October 2015 through April 2016, whereas 98 dried blood samples were collected on filter paper from malaria-suspected patients who visited hospitals or health centres in the northernmost province from November to December 2017. Socio-demographic/behavioural data (age, gender, occupation, education, marital status, religion, ethnicity), as well as bed net use and clinical data (body temperature, any signs and symptoms, history of malaria episodes) are summarized in Additional file 7. In Laos, malaria-suspected patients generally visit the nearest healthcare facility.

Among the 2409 samples from the five southern provinces, 1213 samples were *P. falciparum* positive or mixed *P. falciparum* and *P. vivax* positive. The remaining 1196 samples were composed of 981 *P. vivax* positive, four *Plasmodium malariae* positive, one *Plasmodium ovale* positive, 133 unknown malaria species positive, and 77 *Plasmodium* negative by the PCR. The reason why malaria species could not be identified by the PCR was probably due to some mutations in primer priming sites, or infection of some other *Plasmodium* species, such as simian malaria parasites, whereas the reason why *Plasmodium* negative by the PCR was probably due to false positive results with the RDT, or there being only a small amount of DNA for the PCR amplification. Sixty-two *P. falciparum* samples were excluded from which the *k13* data (41 samples) were failed to obtain or which were shown to have originated from domestic migrants (18 samples) or foreign migrants (three samples). The reason why the *k13* gene was not amplified by the PCR was most probably due to there being only a small amount of DNA for PCR amplification of the *k13*. Finally, 1151 *P. falciparum* samples from the five southern provinces were used for the *k13* analyses. Among the 98 malaria-suspected samples from Phongsaly, the northernmost province, three samples were *P. falciparum* positive, three samples were *P. malariae* positive, and two were *P. vivax* positive. The remaining 90 samples were *Plasmodium* negative by the PCR. The three *P. falciparum* samples from the north were used for the *k13* analyses.

**Fig. 1:**
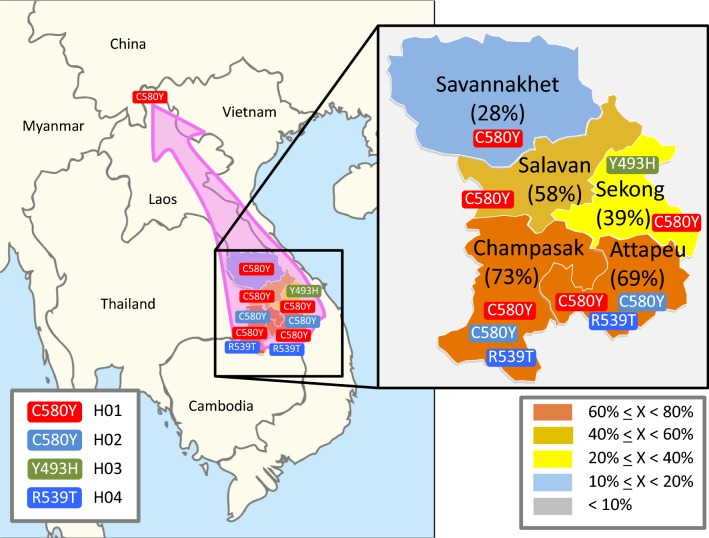
Percentages and types of resistant mutations in the *k13* gene in *Plasmodium falciparum* isolates in five southern provinces. Red with C580Y represents haplotype 1 (H01), light blue with C580Y represents haplotype 2 (H02), light green with Y493H represents haplotype 3 (H03), and blue with R539T represents haplotype 4 (H04). Three resistant isolates of *P. falciparum* were found from the Lao–Chinese border

Overall, 55.5% (639/1151) of the samples from the south possessed non-synonymous mutations in the K13 gene (Table 1). Frequencies of the *k13* mutations in the five provinces were as follows: Savannakhet 28.0% (71/254), Salavan 58.3% (126/216), Sekong 38.6% (54/140), Attapeu 69.1% (85/123), and Champasak 72.5% (303/418) (Table 1 and Fig. 1). There was significant geographic disparity in the frequencies of the *k13* mutations, not only among the provinces, but also within the districts. For example, among the 16 districts in Savannakhet, the frequency of the *k13* mutation in Thapangthong district was the highest (96.6%: 56/58) and that in Nong district was the lowest (2.5%: 3/120) (Table 1).

**Table 1:** Numbers and types of the *k13* mutations in *Plasmodium falciparum* in 16 districts in five southern provinces in Laos

Province	District	No. *P. falciparum* isolates	C580Y	P574L	R539T	Y493H	C580Y/R539T	C580Y/Y493H	Total no. *k13* mutation	Percentage *k13* mutation
Savannakhet	Nong	120	3	0	0	0	0	0	3	2.5
	Phin	37	8	0	0	0	0	0	8	21.6
	Sepon (Xepon)	36	3	0	0	0	0	0	3	8.3
	Thapangthong	58	56	0	0	0	0	0	56	96.6
	Vilabouly	3	1	0	0	0	0	0	1	33.3
	Total	254	71	0	0	0	0	0	71	28.0
Salavan	Taoy	58	8	1	0	0	0	0	9	15.5
	Toumlan	88	53	0	0	0	0	0	53	60.2
	Vapy	70	64	0	0	0	0	0	64	91.4
	Total	216	125	1	0	0	0	0	126	58.3
Sekong	Lamarm	28	1	0	0	10	0	0	11	39.3
	Thateng	112	16	0	0	26	0	1	43	38.4
	Total	140	17	0	0	36	0	1	54	38.6
Attapeu	Phouvong	29	14	0	1	0	0	0	15	51.7
	Sanamxay	91	61	0	6	1	1	0	69	75.8
	Saysetha	3	1	0	0	0	0	0	1	33.3
	Total	123	76	0	7	1	1	0	85	69.1
Champasak	Khong	66	37	0	8	0	1	0	46	69.7
	Mounlapamok	14	9	0	0	0	0	0	9	64.3
	Pathoumphone	338	215	0	29	0	4	0	248	73.4
	Total	418	261	0	37	0	5	0	303	72.5
Total		1151	550	1	44	37	6	1	639	55.5

Sampling period: October 2015–April 2016

Four non-synonymous mutations (C580Y, P574L, R539T, Y493H) were detected (Table 2), of which C580Y, R539T and Y493H were validated for artemisinin-resistance and P574L was reported to be associated with the resistance [3, 16]. The majority of them (632: 98.9%) contained a single mutation and the other 7 (1.1%) contained double mutations (6: C580Y/R539T, 1: C580Y/Y493H) or multiple clone infection. The PCR genotyping of the two gene regions (*msp1* and *msp2*) encoding merozoite surface proteins (MPS1 and MSP2) of *P. falciparum* demonstrated that five of the seven cases were multiple clone infections, while the other two cases were estimated to be double mutations in the *k13* gene (Additional file 8). The dominant mutation, C580Y (557: 87.2%), was observed in all five provinces, as has been commonly found in the other GMS countries [11, 12]. On the other hand, R539T (50: 7.8%), reported from Cambodia, was mainly observed in Champasak (adjacent to Cambodia) [11], and Y493H (38: 5.9%), reported from Cambodia and Vietnam, was mainly observed in Sekong (adjacent to Vietnam) [11]. The three samples from Phongsaly, the northernmost province, also possessed the C580Y mutation in the *k13* gene.

**Table 2:** Numbers and types of the *k13* mutations in *Plasmodium falciparum* in provinces by year, gender and occupation

Status	Total	C580Y	P574L	R539T	Y493H	Chi-test
Province						
Total	632	550	1	44	37	< 0.001
Savannaket	71	71	0	0	0	
Salavan	126	125	1	0	0	
Sekong	53	17	0	0	36	
Attapeu	84	76	0	7	1	
Champasak	298	261	0	37	0	
Year						
Total	632	550	1	44	37	0.001
Oct.–Dec. 2015	302	272	0	24	6	
Jan.–Apr. 2016	330	278	1	20	31	
Gender						
Total	632	550	1	44	37	0.006
Male	580	503	0	42	35	
Female	52	47	1	2	2	
Occupation						
Total	632	550	1	44	37	0.048
Other	108	91	1	6	10	
Soldier/agriculture	524	459	0	38	27	

Data of the *P. falciparum* isolates possessing single mutation were shown

The genotypes of the two flanking loci (K13_151 and K13_159) of the *k13* gene were determined for 438 isolates (average 87.6 isolates/province) in the five southern provinces. These two loci were highly polymorphic with insertion/deletion and SNPs. The K13_151 possessed seven alleles and the K13_159 possessed 16 alleles. A total of 37 flanking haplotypes were observed among them (Additional files 5, 6). The most predominant haplotype H01 with the C580Y mutation in the *k13* gene (39.7%: 174/438) was observed from all five provinces, whereas the second predominant haplotype H02 with the C580Y mutation (9.6%: 42/438) was observed mainly from the two southern provinces (Attapeu, Champasak). The third predominant haplotype H03 was found in the isolates with the Y493H mutation (7.5%: 33/438) from Sekong, which is adjacent to Vietnam. The three *P. falciparum* from Phongsaly, northernmost province, also possessed the C580Y mutation with the haplotype H01 (Additional files 5, 6).

Results of bivariate analyses to estimate an association between variables and contracting *P. falciparum* possessing the *k13* mutation from the five southern provinces are shown in Additional file 7. Gender balance (proportion between male and female) and age distribution of the participants showed no difference among the five southern provinces. Risk factors associated with contracting *P. falciparum* possessing the *k13* mutation were estimated by multivariate logistic regression analyses. Four variants were independently associated with contracting *P. falciparum* possessing the *k13* mutation: residing in Attapeu and Champasak (Model 1, adjusted odds ratio (AOR) 1.57, 95% CI 1.14–2.16), adult (Model 1, AOR 2.24, 95% CI 1.18–4.22), male (Model 1, AOR 1.96, 95% CI 1.32–2.90), and malaria history (≥ 3 times) (Model 1, AOR 1.59, 95% CI 1.15–2.21) (Table 3). In other words, regardless of the residential area, adult male with at least three episodes of malaria had significantly higher risk to get *P. falciparum* harbouring the *k13* mutation.

**Table 3:** Multivariate logistic regression analyses to identify risk factors for contracting the *k13* mutated *Plasmodium falciparum*

Status	OR	95% CI	*P*	Model 1			Model 2		
				AOR	95% CI	*P*	AOR	95% CI	*P*
Province									
Other	1.00			1.00			1.00		
Attapeu and Champasak	3.63	2.83–4.64	< 0.001	1.57	1.14–2.16	0.006	2.57	1.98–3.32	< 0.001
Age									
Child	1.00			1.00			1.00		
Adult	7.5	4.39–12.8	< 0.001	2.24	1.18–4.22	0.013	3.49	1.97–6.18	< 0.001
Gender									
Female	1.00			1.00			1.00		
Male	4.17	2.95–5.88	< 0.001	1.96	1.32–2.90	0.001	2.21	1.51–3.22	< 0.001
Occupation									
Other	1.00			1.00			–		
Soldier/agriculture	2.95	2.24–3.88	< 0.001	1.15	0.80–1.65	0.451	–	–	–
Education									
Less	1.00			1.00			–		
≥ High school	2.16	1.29–3.62	0.004	1.53	0.87–2.70	0.141	–	–	–
Religion									
Not buddhism	1.00			1.00			–		
Buddhism	4.70	3.64–6.05	< 0.001	1.29	0.78–2.14	0.330	–	–	–
Ethnicity									
Lao Theung	1.00			1.00			–		
Lao	4.89	3.80–6.29	< 0.001	2.09	1.25–3.48	0.005	–	–	–
Start symptom									
Other	1.00			1.00			–		
Today	1.65	0.97–2.79	0.063	1.61	0.91–2.84	0.101	–	–	–
Malaria history									
Fewer	1.00			1.00			1.00		
≥ 3 times	2.53	1.87–3.41	< 0.001	1.59	1.15–2.21	0.006	1.77	1.29–2.44	< 0.001
Bed-net									
No	1.00			1.00			–		
Yes	1.28	0.99–1.28	0.061	0.95	0.74–1.33	0.971	–	–	–

*OR* odds ratio, *CI* confidence interval, *AOR* adjusted odds ratio

## Discussion

In this study, to understand the current situation of artemisinin-resistant *P. falciparum* in malaria endemic areas in Laos, the frequencies of the *k13* mutations were determined in the 1151 field isolates of *P. falciparum* from the five southern provinces in Laos from October 2015 to April 2016 and those in the three isolates from the northernmost province in November to December 2017. Generally, a history of anti-malarial drug usage in the endemic area or of each patient are important factors to explain the emergence and spread of the drug resistance. Artemether–lumefantrine, one of the artemisinin-based combinations, has been used in Laos as the first-line treatment regimen for *P. falciparum* since 2005 and for *P. vivax* since 2011 [17]. Through the support of the Global Fund, the ACT is prescribed to malaria patients free of charge at public healthcare facilities, and some private clinics and pharmacies after a confirmation of *Plasmodium* infection by microscopy or RDTs, which is termed “Private–Public Mix programme” [18]. Therefore, currently, the ACT is not available without proper diagnosis. However, in Laos, artemisinin mono-therapy had been available until it was banned in 2008. There is a paucity of detailed information about the usage of the artemisinin mono-therapy [17]. On the other hand, chloroquine is still freely available without diagnosis or prescription. According to the result of the questionnaire survey in this study, only 18 out of the 1151 patients took anti-malarial drugs before visiting the public healthcare facilities. Seven of the 18 patients took chloroquine, four took artesunate mono-therapy, one took primaquine, one took quinine and the information of anti-malarial drug usage of the other five patients was not obtained. Anti-malarial drug usage of the patients before visiting the healthcare facilities in this study, did not affect the results of the multiple variate analysis to estimate the risk factors to contract with *P. falciparum* isolates with the *k13* mutation.

The extreme difference of false positivity rates between the samples from the south and the north is due to the sampling methods. In the south, the dried blood samples were collected only form malaria patients confirmed by microscopy or the RDT, while in the north, the samples were collected from both positive and negative patients by microscopy or the RDT. In addition, in the northernmost province, *P. vivax* was the predominant *Plasmodium* species and the prevalence of *P. falciparum* was relatively low during the sampling period of this study.

Although the frequencies of the *k13* mutations in each province were heterogeneous, there was a tendency for the frequency to be highest in the southernmost provinces of the country (Champasak, Attapeu) and gradually decreasing toward the north (Salavan, Sekong, Savannakhet). These observed haplotype distribution findings suggest that one of the lineages of artemisinin-resistant *P. falciparum* with the C580Y mutation (H01) has spread from southern to northern Laos, and some other artemisinin-resistant lineages (H02, H03, H04, and others) have only spread within more limited areas. For example, the haplotype H03 was mainly found in the isolates with the Y493H mutation (7.5%: 33/438) in Sekong province, which is adjacent to Vietnam. Indeed, the previous study reported that the Y493H mutation was observed in southern part of Vietnam [11]. This suggests that the lineages with the Y493H mutation in Laos and Vietnam probably share a common ancestor.

According to a study on microsatellite haplotypes flanking the *k13* gene among the field isolates of *P. falciparum* from Cambodia, Thailand and Laos, a single lineage of artemisinin-resistant *P. falciparum* with the C580Y mutation in the *k13* gene was suggested to have spread from western parts of Cambodia to southeastern parts of Thailand, and then to Champasak in Laos [12]. Moreover, in a very recent study, the analysis of the C580Y mutation in the *k13* gene and flanking microsatellite loci demonstrated that the lineage originating from western Cambodia (Pailin) has spread to the southern part of Vietnam [19]. The H01 is identical to the Pailin isolate with the C580Y mutation in the previous study with the exception of a single base pair deletion in the 151 locus [11]. These reported dissemination patterns, together with findings in the present study, demonstrate that the resistant isolates with the C580Y mutation have most likely been introduced from Thailand/Cambodia and subsequently spread through southern Laos, but have also taken a sharp northward direction in Laos. On the other hand, when the frequencies of the *k13* mutations in each district of the five southern provinces were observed, the situation seems to be more complicated. Indeed, the frequencies of the *k13* mutations were relatively high in the districts of Champasak province but not the highest (Table 1, Additional file 9). Surprisingly, an extremely high frequency of the C580Y mutation in the *k13* gene was observed in Thapangthong district (96.6%), Savannakhet province and an almost equally high frequency was also observed in Vapy district (91.4%), Salavan province, which is directly adjacent to Thapangthong district (Table 1, Additional file 9). According to Savannakhet Provincial Health Department, logging was very active in the two districts during the sampling period. Moreover, several studies have reported that forest workers, such as logging and mining personnel, were high-risk groups for malaria infection [20–29]. In fact, a previous study from Thapangthong district in 2015 already showed a high frequency of the C580Y mutation in the *k13* gene (75%) [30]. Even within a single province, the frequencies of the *k13* mutations were not the same among the districts. For example, in Nong district in Savannakhet province, which is adjacent to Vietnam, the frequency was much lower (2.5%) than that in Thapangthong district (96.6%). On the contrary, in the southernmost province, Champasak, the frequencies of the *k13* mutations were high (> 64%) among the three districts. The situation in Attapeu province was also similar to that in Champasak province. The frequencies of the *k13* mutations in two of the three districts (Phouvong and Sanamxay) in Attapeu province were also high (> 52%). The frequency in the remaining district (Saysetha) was relatively low (33%) but only three isolates were examined from that district in the present study. These results suggest that the frequencies of the *k13* mutations are high in the south and west, whereas relatively low in the east of the southern part of Laos.

Risk factors for contracting *P. falciparum* with the *k13* mutations were: male, adult, Lao (major ethnicity), residing in Champasak and Attapeu provinces, and a history of malaria episodes (≥ 3 times). These findings, especially being male and adult, suggest that *P. falciparum* strains transmitting among adult males were different from those transmitting among children and females as inferred from the results. Fewer artemisinin-resistant *P. falciparum* strains were transmitting in the villages, whereas more artemisinin-resistant *P. falciparum* strains were transmitting outside the villages, such as in the fields and the forests where adult males work and sometimes stay overnight without appropriate preventive measures against mosquitoes (Table 3; Additional file 7). Why Lao, the major ethnic group (Lao living in the lowlands), had a higher risk of contracting *P. falciparum* with the *k13* mutations compared to Lao Theung (Lao living in midlands) remains unknown.

Laos is a land-locked country, but recently it is termed a land-linked country due to substantial improvement of road conditions and economic development [31, 32]. Additionally, the ASEAN Economic Community was officially launched in late 2015, which has further accelerated economic activities among the peoples in member countries, including Laos. Such economic development may also enhance the spread of malaria, including drug-resistant malaria, by movement of both Laotians and non-Laotians between countries in the GMS.

Malaria outbreaks were reported among ‘mobile and migrant workers’ in Laos [2]. In 2011 in Attapeu province, a malaria outbreak occurred among the workers of a hydropower dam project. Approximately 70% of confirmed malaria cases in southern provinces were both from other provinces in Laos and neighbouring countries [2]. Furthermore, outbreak investigations by the Lao Ministry of Health revealed that most focal outbreaks during 2011 occurred in the central and northern provinces, whereas outbreaks in 2015 were due to resident mobile workers in northern provinces who were returning from the endemic southern provinces of Attapeu, Champasak, Sekong, and Salavan [2].

Monitoring the northern limit of the prevalence of artemisinin-resistant malaria in Laos is an urgent issue for containment of the resistance within the country. *Plasmodium* sample collections were started and analyses in November 2017 in the northernmost province of the country, Phongsaly, which has a common border with Yunnan province, China. Confirming the hypothesis, three *P. falciparum* isolates with the C580Y mutation were discovered in December 2017 from malaria patients. These patients were living in Gnot-Ou district (bordering China), Phongsaly province, who had not received a blood transfusion. Neither they nor their family members had any travel histories outside the province. In addition, the flanking haplotype analysis of these resistant isolates proved that the isolates were the H01 that is the predominant isolate in the southern part of Laos. These findings suggest that some malaria patients and/or asymptomatic *Plasmodium* carriers who had contracted artemisinin-resistant *P. falciparum* isolates in the southern provinces came to the northernmost province, and that their parasites were locally transmitted by *Anopheles* mosquitos in the northernmost province. Moreover, the haplotype analysis in this study also proved that the H01 was genetically very close to the *P. falciparum* isolate with the C580Y mutation from Pailin, the western part of Cambodia. This evidence strongly suggests that the artemisinin-resistant strain of *P. falciparum* originating from western Cambodia has already reached the Lao–Chinese border. Presently, Chinese migrant workers are coming to Laos more and more for the railway construction between China and Laos near Phongsaly and in other northern provinces. Therefore, malaria prevention is needed not only for Laotians, but also for the Chinese migrant workers in the northern part of Laos for the containment the artemisinin-resistant malaria within the country.

## Conclusions

Artemisinin-resistant *P. falciparum* was rapidly increasing and spreading northwards through Laos and has already reached Phongsaly, the northernmost province adjacent to Yunnan province, China. Further investigation is needed to clarify the artemisinin-resistant malaria situation in Phongsaly province and other northern provinces in Laos, as well as in Yunnan province in China. It should be noted that, recently, the F446I mutation in the *k13* gene, which is associated with delayed parasite clearance, has already been identified from Yunnan province, China adjacent to Myanmar [33, 34]. This alarming situation raises major concerns for the containment of artemisinin-resistant strains within Laos as well as the GMS.

## Additional files

**Additional file 1.** Public healthcare facilities studied in five southern provinces, Lao PDR.

**Additional file 2.** Experimental procedures.

**Additional file 3.** PCR primers, master mix of RCR and condition of assay.

**Additional file 4.** Nucleotide sequence alignment of PF3D7_1337500 (K13_151) and PF3D7_1339700 (K13_159) alleles, located 200 Kb upstream from *k13* on chromosome 13, found in 441 isolates of *Plasmodium falciparum* from the five southern provinces (n = 438) and one northern province (n = 3) in Laos.

**Additional file 5.** Haplotypes based on the two flanking loci of the *k13* gene in the six provinces, summarized by provinces.

**Additional file 6.** Haplotypes based on the two flanking loci of the *k13* gene in the six provinces, summarized by frequency of the haplotypes.

**Additional file 7.** Bivariate analyses to identify an association between variables and *Plasmodium falciparum* infections with the *k13* mutations in the five provinces.

**Additional file 8.** PCR genotyping based on *msp1* and *msp2.*

**Additional file 9.** Frequencies of the *k13* mutations in the districts.

## Abbreviations

GMS: The Greater Mekong Sub-region
ACT: artemisinin-based combination therapy
Lao PDR: Lao People’s Democratic Republic
*k13*: *kelch*-*propeller* domains in chromosome 13 in *P. falciparum*
