# Supplementary material for: Heterogeneous distribution of k13 mutations in Plasmodium falciparum in Laos
Source: Malar J. 2018 Dec 27;17:483. doi: 10.1186/s12936-018-2625-6 (PMC6307170; doi:10.1186/s12936-018-2625-6)
Supplement: Supplementary file 1 — Additional file 1. Public healthcare facilities studied in five southern provinces, Lao PDR. [file 12936_2018_2625_MOESM1_ESM.docx]

**Additional File 1**  List of public healthcare facilities studied in five southern provinces and one northernmost province, Lao PDR

| No. | Province | District (API) | Name of Public Health Facility |
| --- | --- | --- | --- |
| 1 | Savannakhet | Kaysone Phomvihane (0.45) | Malaria section, Savannakhet Provincial Health Office |
| 2 | Savannakhet | Nong (20.7) | Malaria section, Nong District Health Office |
| 3 | Savannakhet | Nong (20.7) | Nong District Hospital |
| 4 | Savannakhet | Nong (20.7) | Dongnasarn Health Center |
| 5 | Savannakhet | Nong (20.7) | Daenvilay Health Center |
| 6 | Savannakhet | Nong (20.7) | Asing Health Center |
| 7 | Savannakhet | Nong (20.7) | Nakong Health Center |
| 8 | Savannakhet | Nong (20.7) | Phounmarkmee Health Center |
| 9 | Savannakhet | Nong (20.7) | Tong alai Health Center |
| 10 | Savannakhet | Nong (20.7) | Kaisone Health Center |
| 11 | Savannakhet | Nong (20.7) | Lakai Health Center |
| 12 | Savannakhet | Phin (17.5) | Malaria section, Phin District Health Office |
| 13 | Savannakhet | Phin (17.5) | Phin District Hospital |
| 14 | Savannakhet | Phin (17.5) | Huayhoy Health Center |
| 15 | Savannakhet | Phin (17.5) | Yang Health Center |
| 16 | Savannakhet | Phin (17.5) | Nathom Health Center |
| 17 | Savannakhet | Phin (17.5) | Sethamuak Health Center |
| 18 | Savannakhet | Phin (17.5) | Nathong Health Center |
| 19 | Savannakhet | Phin (17.5) | Hin Sangom Health Center |
| 20 | Savannakhet | Phin (17.5) | Tad Hai Health Center |
| 21 | Savannakhet | Sepon (15.8) | Malaria section, Sepon District Health Office |
| 22 | Savannakhet | Sepon (15.8) | Sepon District Hospital |
| 23 | Savannakhet | Sepon (15.8) | Dongsavan Health Center |
| 24 | Savannakhet | Sepon (15.8) | Lardhor Health Center |
| 25 | Savannakhet | Sepon (15.8) | Lako Health Center |
| 26 | Savannakhet | Sepon (15.8) | Lavern Health Center |
| 27 | Savannakhet | Sepon (15.8) | Kapaiy Health Center |
| 28 | Savannakhet | Sepon (15.8) | Phonhai Health Center |
| 29 | Savannakhet | Sepon (15.8) | Daensavan Health Center |
| 30 | Savannakhet | Sepon (15.8) | Katoub Health Center |
| 31 | Savannakhet | Sepon (15.8) | Manjee Health Center |
| 32 | Savannakhet | Sepon (15.8) | Sobmy Health Center |
| 33 | Savannakhet | Sepon (15.8) | LaArn Health Center |
| 34 | Savannakhet | Sepon (15.8) | Phabang Health Center |
| 35 | Savannakhet | Thapangthong (49.6) | Malaria section, Thapangthong District Health Office |
| 36 | Savannakhet | Thapangthong (49.6) | Thapangthong District Hospital |
| 37 | Savannakhet | Thapangthong (49.6) | Phumaly Health Center |
| 38 | Savannakhet | Thapangthong (49.6) | Seuak Health Center |
| 39 | Savannakhet | Thapangthong (49.6) | Thaphy Health Center |
| 40 | Savannakhet | Thapangthong (49.6) | Sepong Health Center |
| 41 | Savannakhet | Thapangthong (49.6) | Houy Meun Health Center |
| 42 | Savannakhet | Thapangthong (49.6) | Kathongneua Health Center |
| 43 | Savannakhet | Vilabouly (15.4) | Malaria section, Vilabouly District Health Office |
| 44 | Savannakhet | Vilabouly (15.4) | Vilabouly District Hospital |
| 45 | Savannakhet | Vilabouly (15.4) | Nampadan Health Center |
| 46 | Savannakhet | Vilabouly (15.4) | Dongyarng Health Center |
| 47 | Savannakhet | Vilabouly (15.4) | Nayom Health Center |
| 48 | Savannakhet | Vilabouly (15.4) | Nasienglae Health Center |
| 49 | Savannakhet | Vilabouly (15.4) | Angkham Health Center |
| 50 | Savannakhet | Vilabouly (15.4) | Nammahy Health Center |
| 51 | Savannakhet | Vilabouly (15.4) | Meungvang Health Center |
| 52 | Savannakhet | Vilabouly (15.4) | Nakae Health Center |
| 53 | Salavan | Salavan (6.9) | Malaria section, Salavan Provincial Health Office |
| 54 | Salavan | Taoy (51.4) | Malaria section, Taoy District Health Office |
| 55 | Salavan | Taoy (51.4) | Taoy District Hospital |
| 56 | Salavan | Taoy (51.4) | Toomlythong Health Center |
| 57 | Salavan | Taoy (51.4) | Ta Huak Health Center |
| 58 | Salavan | Taoy (51.4) | Kok Bok Health Center |
| 59 | Salavan | Taoy (51.4) | Thong Ka Hai Health Center |
| 60 | Salavan | Taoy (51.4) | Pachoudone Health Center |
| 61 | Salavan | Taoy (51.4) | Phortang Health Center |
| 62 | Salavan | Taoy (51.4) | Bongnam Health Center |
| 63 | Salavan | Taoy (51.4) | Huay Ngua Health Center |
| 64 | Salavan | Toumlan (113.1) | Malaria section, Toumlan District Health Office |
| 65 | Salavan | Toumlan (113.1) | Toumlan District Hospital |
| 66 | Salavan | Toumlan (113.1) | Snow Health Center |
| 67 | Salavan | Toumlan (113.1) | Tambeng Health Center |
| 68 | Salavan | Toumlan (113.1) | Nadou Health Center |
| 69 | Salavan | Toumlan (113.1) | Taeyor Health Center |
| 70 | Salavan | Toumlan (113.1) | Thamuang (Kokmuang) Health Center |
| 71 | Salavan | Toumlan (113.1) | Donkhayoug Health Center |
| 72 | Salavan | Vapy (68.9) | Malaria section, Vapy District Health Office |
| 73 | Salavan | Vapy (68.9) | Vapy District Hospital |
| 74 | Salavan | Vapy (68.9) | Huay Khon Health Center |
| 75 | Salavan | Vapy (68.9) | Khonesaiy Health Center |
| 76 | Salavan | Vapy (68.9) | Nalan Health Center |
| 77 | Salavan | Vapy (68.9) | Nasied Health Center |
| 78 | Salavan | Vapy (68.9) | Sphard Health Center |
| 79 | Sekong | Lamarn (22.9) | Malaria section, Sekong Provincial Health Office |
| 80 | Sekong | Lamarn (22.9) | Sekong Provincial Hospital |
| 81 | Sekong | Lamarn (22.9) | Ban Navay Health Center |
| 82 | Sekong | Lamarn (22.9) | Tok Ong Keo Health Center |
| 83 | Sekong | Lamarn (22.9) | Kasang Kang Health Center |
| 84 | Sekong | Lamarn (22.9) | Torksaming Health Center |
| 85 | Sekong | Lamarn (22.9) | Donchan Health Center |
| 86 | Sekong | Lamarn (22.9) | Ban Phon Health Center |
| 87 | Sekong | Lamarn (22.9) | Ta neum Health Center |
| 88 | Sekong | Thateng (8.7) | Malaria section, Thateng District Health Office |
| 89 | Sekong | Thateng (8.7) | Thateng District Hospital |
| 90 | Sekong | Thateng (8.7) | Huaydam Health Center |
| 91 | Sekong | Thateng (8.7) | Kokphoung Health Center |
| 92 | Sekong | Thateng (8.7) | Chounla Health Center |
| 93 | Sekong | Thateng (8.7) | Nong Nok Health Center |
| 94 | Sekong | Thateng (8.7) | Thon Noy Health Center |
| 95 | Sekong | Thateng (8.7) | Thongwai Health Center |
| 96 | Sekong | Thateng (8.7) | Yeub Health Center |
| 97 | Attapeu | Samakkhixay (16.8) | Malaria section, Attapeu Provincial Health Office |
| 98 | Attapeu | Phouvong (103.4) | Malaria section, Phouvong District Health Office |
| 99 | Attapeu | Phouvong (103.4) | Phouvong District Hospital |
| 100 | Attapeu | Phouvong (103.4) | Naseuk (Xeuak) Health Center |
| 101 | Attapeu | Phouvong (103.4) | Lamong Health Center |
| 102 | Attapeu | Phouvong (103.4) | Phoukeau Health Center |
| 103 | Attapeu | Phouvong (103.4) | Phouhome Health Center |
| 104 | Attapeu | Phouvong (103.4) | Somboun Health Center |
| 105 | Attapeu | Phouvong (103.4) | Viengxay Health Center |
| 106 | Attapeu | Sanamxay (59.9) | Malaria section, Sanamxay District Health Office |
| 107 | Attapeu | Sanamxay (59.9) | Sanamxay District Hospital |
| 108 | Attapeu | Sanamxay (59.9) | Ban Mai Health Center |
| 109 | Attapeu | Sanamxay (59.9) | Banthae Health Center |
| 110 | Attapeu | Sanamxay (59.9) | Bengvilay Health Center |
| 111 | Attapeu | Sanamxay (59.9) | Park Bo Health Center |
| 112 | Attapeu | Sanamxay (59.9) | Sompoi Health Center |
| 113 | Attapeu | Sanamxay (59.9) | Khang Health Center |
| 114 | Attapeu | Sanamxay (59.9) | Oudomsouk Health Center |
| 115 | Attapeu | Sanamxay (59.9) | Xaidonekhong Health Center |
| 116 | Attapeu | Saysetha (31.2) | Malaria section, Saysetha District Health Office |
| 117 | Attapeu | Saysetha (31.2) | Saysetha District Hospital |
| 118 | Attapeu | Saysetha (31.2) | Keng Yai Health Center |
| 119 | Attapeu | Saysetha (31.2) | Sakae Health Center |
| 120 | Attapeu | Saysetha (31.2) | Sapuan Health Center |
| 121 | Attapeu | Saysetha (31.2) | Wat Neua Health Center |
| 122 | Attapeu | Saysetha (31.2) | Yai Oudom (Ban Markeau) Health Center |
| 123 | Champasak | Pakse (0.02) | Malaria section, Champasak Provincial Health Office |
| 124 | Champasak | Khong (35.0) | Malaria section, Khong District Health Office |
| 125 | Champasak | Khong (35.0) | Khong District Hospital |
| 126 | Champasak | Khong (35.0) | Ban Sod Health Center |
| 127 | Champasak | Khong (35.0) | Nafang Health Center |
| 128 | Champasak | Khong (35.0) | Phonsa Ard Health Center |
| 129 | Champasak | Khong (35.0) | Nakasang Health Center |
| 130 | Champasak | Khong (35.0) | Donetharn Health Center |
| 131 | Champasak | Khong (35.0) | Khone Health Center |
| 132 | Champasak | Khong (35.0) | Lobparkdy Health Center |
| 133 | Champasak | Khong (35.0) | Donesoam Health Center |
| 134 | Champasak | Khong (35.0) | Kynark Health Center |
| 135 | Champasak | Khong (35.0) | Ban Boung Health Center |
| 136 | Champasak | Khong (35.0) | Huakhong Health Center |
| 137 | Champasak | Khong (35.0) | Hythard Health Center |
| 138 | Champasak | Mounlapamok (36.9) | Malaria section, Mounlapamok District Health Office |
| 139 | Champasak | Mounlapamok (36.9) | Mounlapamok District Hospital |
| 140 | Champasak | Mounlapamok (36.9) | Nady Health Center |
| 141 | Champasak | Mounlapamok (36.9) | Thahae Health Center |
| 142 | Champasak | Mounlapamok (36.9) | Vernyarng Health Center |
| 143 | Champasak | Mounlapamok (36.9) | Nong Nga Health Center |
| 144 | Champasak | Mounlapamok (36.9) | Kadan Health Center |
| 145 | Champasak | Mounlapamok (36.9) | Nonghoy Health Center |
| 146 | Champasak | Pathoumphone (53.8) | Malaria section, Pathoumphone District Health Office |
| 147 | Champasak | Pathoumphone (53.8) | Pathoumphone District Hospital |
| 148 | Champasak | Pathoumphone (53.8) | Kaelae Health Center |
| 149 | Champasak | Pathoumphone (53.8) | Pathoumphone Health Center |
| 150 | Champasak | Pathoumphone (53.8) | Phapho Health Center |
| 151 | Champasak | Pathoumphone (53.8) | Sanod (Ban Non) Health Center |
| 152 | Champasak | Pathoumphone (53.8) | Nongpakhed Health Center |
| 153 | Champasak | Pathoumphone (53.8) | Ban Boum Health Center |
| 154 | Champasak | Pathoumphone (53.8) | Donedeng Health Center |
| 155 | Champasak | Pathoumphone (53.8) | Km 24 Health Center |
| 156 | Phongsaly | Phongsaly (0.86) | Malaria section, Phongsaly Provincial Health Office |
| 157 | Phongsaly | Boun Neua (1.64) | Phongsaly Provincial Hospital |
| 158 | Phongsaly | Gnot Ou (1.82) | Malaria sextion, Gnot Ou District Health Office |
| 159 | Phongsaly | Gnot Ou (1.82) | Gnot Ou District Hospital |
| 160 | Phongsaly | Gnot Ou (1.82) | Bang Tang Health Center |
| 161 | Phongsaly | Gnot Ou (1.82) | Ban Ou Neau Health Center |
| 162 | Phongsaly | Gnot Ou (1.82) | Saew Chai Health Center |
| 163 | Phongsaly | Gnot Ou (1.82) | Malythao Health Center |

API: annual parasite incidence per 1,000 population (2013), Lao Ministry of Health.
