# Supplementary material for: Heterogeneous distribution of k13 mutations in Plasmodium falciparum in Laos
Source: Malar J. 2018 Dec 27;17:483. doi: 10.1186/s12936-018-2625-6 (PMC6307170; doi:10.1186/s12936-018-2625-6)
Supplement: Supplementary file 2 — Additional file 2. Experimental procedures. [file 12936_2018_2625_MOESM2_ESM.docx]

**Additional File 2** Experimental procedures

**DNA extraction**

DNA was extracted from the dried blood spots on the filter papers with a Maxwell® RSC Instrument (Promega, Madison WI, USA) in accordance with the manufacturer’s instructions with minor modification. Briefly, 3 punched-out circles (3.175 mm diameter [1/8 inch]) from the dried blood spot on the filter paper were used for DNA extraction, which was equivalent to 15–20 µL of whole blood. The punched-out filter paper circles were incubated in 30 µL of proteinase-K and 180 µL of incubation buffer from the kit at 70o C for 90 minutes and then followed with the extraction instructions. The extracted DNA was eluted with 50 µL of elution buffer and preserved until use at -30°C.

***Plasmodium* species identification by PCR**

To identify *Plasmodium* species, a real-time PCR was conducted using primer sets that were designed in the present study and in a previous study (Table S2). For the real-time PCR, specific primer sets for amplifying the partial *cytochrome b* gene on the mitochondrial genome of *P. falciparum* and *P. vivax* were used. When *P. falciparum* or *P. vivax* DNA was not detected by those primer sets, *P. malariae* and *P. ovale* specific primer sets were also used for the PCR. The real-time PCR was performed using SsoAdvanced™ Universal SYBR® Green Supermix (Bio-Rad Laboratories, Inc., USA) using 2 µL of the extracted DNA as a template, which was equivalent to 0.6–0.8 µL of whole blood. Serial diluted recombinant plasmid DNAs containing the *cytochrome b* region of each species were used as the positive controls for each assay. To monitor cross-contamination, PCR-grade water was used as the negative control for each assay. A sample was considered negative if there was no increase in the SYBR® Green (fluorescent) signal after 35 cycles.

**DNA analysis of the K13 mutations**

Mutations in the *K13* gene were examined by the K13 toolbox previously described by Ménard, et al., 2016. When the *K13* gene region was not amplified by the primer sets in the K13 toolbox, additional primer sets (K13FF, K13RR, NF11 and NR22) were designed and used (Table S2). The PCR products were purified with Performa DTR Gel Filtration Cartridge (Edge Bio, Gaithersburg, MD, USA) and sequenced by an ABI Genetic Analyzer model 3130XL (Thermo Fisher Scientific, Tokyo, Japan). When the mutation was observed in the K13 gene in the sample, we performed the PCR amplification and DNA sequencing once again as an internal quality assessment. DNA of the FCR-3 strain of *P. falciparum* was used as a positive control and PCR-grade water was used as the negative control of the *K13* PCR. The sequencing results were analyzed by ClustalW in MEGA version 7.0.21 (Kumar, et al., 2016) using the 3D7 kelch13 sequence as a reference (Accession: XM_001359122.1). The samples with mixed alleles (wild type and mutated type) were considered to be mutated isolates to estimate the frequency of the *K13* mutations.

**DNA analysis of two flanking loci of the *K13* gene, PF3D7_1337500 (K13_151) and PF3D7_1339700 (K13_159)**

Two flanking loci of the *K13* gene, PF3D7_1337500 (K13_151) and PF3D7_1339700 (K13_159), were examined by a method previously described by Ménard, et al., 2016. When the K13_159 locus was not amplified by the primer sets in the method by Ménard, additional primers (159 F1 and 159 R4) were designed and used (Table S2). The PCR products were purified with Performa DTR Gel Filtration Cartridge (Edge Bio, Gaithersburg, MD, USA) and sequenced by an ABI Genetic Analyzer model 3130XL (Thermo Fisher Scientific, Tokyo, Japan). Haplotypes were generated based on the combinations of the two loci to assess dissemination of the K13 mutations (Table S3 and S4). The sequencing results were analyzed by Clustal W in MEGA version 7.0.21 (Kumar, et al., 2016) using PF3D7_1337500 (K13_151) and PF3D7_1339700 (K13_159) in the 3D7 sequence as a reference (Accession: XM_001359122.1).
