# Supplementary material for: Heterogeneous distribution of k13 mutations in Plasmodium falciparum in Laos
Source: Malar J. 2018 Dec 27;17:483. doi: 10.1186/s12936-018-2625-6 (PMC6307170; doi:10.1186/s12936-018-2625-6)
Supplement: Supplementary file 3 — Additional file 3. PCR primers, master mix of RCR and condition of assay. [file 12936_2018_2625_MOESM3_ESM.docx]

**Additional File 3**  PCR primers, master mix of RCR and condition of assay

**Reference**

Ménard D, Khim N, Beghain J, et al. A Worldwide Map of *Plasmodium falciparum* K13-Propeller Polymorphisms. N Engl J Med 2016;374:2453-64.

Putaporntip C, Buppan P, Jongwutiwes S. Improved performance with saliva and urine as alternative DNA sources for malaria diagnosis by mitochondrial DNA-based PCR assays. Clin Microbiol Infect.2011;17:1484-91.

Kumar S, Stecher G, Tamura K. MEGA7: Molecular Evolutionary Genetics Analysis Version 7.0 for Bigger Datasets. Mol Biol Evol. 2016;33(7):1870-4.
