# Supplementary material for: Heterogeneous distribution of k13 mutations in Plasmodium falciparum in Laos
Source: Malar J. 2018 Dec 27;17:483. doi: 10.1186/s12936-018-2625-6 (PMC6307170; doi:10.1186/s12936-018-2625-6)
Supplement: Supplementary file 4 — Additional file 4. Nucleotide sequence alignment of PF3D7_1337500 (K13_151) and PF3D7_1339700 (K13_159) alleles, located 200 Kb upstream from k13 on chromosome 13, found in 441 isolates of Plasmodium falciparum from the five southern provinces (n = 438) and one northern province (n = 3) in Laos. [file 12936_2018_2625_MOESM4_ESM.docx]

**Additional File 4** Nucleotide sequence alignment of PF3D7_1337500 (K13_151) and PF3D7_1339700 (K13_159) alleles, located 200Kb upstream from *k13* on chromosome 13, found in 441 isolates of *Plasmodium falciparum* from the five southern provinces (n=438) and one northern province (n=3) in Laos

**K13_151 alleles**

151_G1(3D7) TTATTATTATTATCATTATTATTATATATATTGTTGTTATTATATATATTGCTGTTATTA [ 60]

151_G2 TTATTATTATTATCATTATTATTATATATATTGTTGTTATTATATATATTGCTGTTATTA [ 60]

151_G3 TTATTATTATTATCATTATTATTATATATATTGTTGTTATTATATATATTGCTGTTATTA [ 60]

151_G4 TTATTATTATTATCATTATTATTATATATATTGTTGTTATTATATATATTGCTGTTATTA [ 60]

151_G5 TTATTATTATTATCATTATTATTATATATATTGTTGTTATTATATATATTGCTGTTATTA [ 60]

151_G6 TTATTATTATTATCATTATTATTATATATATTGTTGTTATTATATATATTGCTGTTATTA [ 60]

151_G7 TTATTATTATTATCATTATTATTATATATATTGTTGTTATTATATATATTGCTGTTATTA [ 60]

151_G1(3D7) TATATATTGTTATTATTATATATATTGCTGTTATTATATATATTATCATTATTATATATA [120]

151_G2 TATATATTGTTATTATTATATATATTGCTGTTATTATATATATTATCATTATTATATATA [120]

151_G3 TATATATTGTTATTATTATATATATTGCTGTTATTATATATATTATCATTATTATATATA [120]

151_G4 TATATATTGTTATTATTATATATATTGCTGTTATTATATATATTATCATTATTATATATA [120]

151_G5 TATATATTGTTATTATTATATATATTGCTGTTATTATATATATTATCATTATTATATATA [120]

151_G6 TATATATTGTTATTATTATATATATTGCTGTTATTATATATATTATCATTATTATATATA [120]

151_G7 TATATATTGTTATTATTATATATATTGCTGTTATTATATATATTATCATTATTATATATA [120]

151_G1(3D7) TTGTTGTTATATATATTATCATTATTATATATAGTATCATTATTATATATATTGTTGTTA [180]

151_G2 TTGTTGTTATATATATTATCATTATTATATATAGTATCATTATTATATATATTGTTGTTA [180]

151_G3 TTGTTGTTATATATATTATCATTATTATATATAGTATCATTATTATATATATTGTTGTTA [180]

151_G4 TTGTTGTTATATATATTATCATTATTATATATAGTATCATTATTATATATATTGTTGTTA [180]

151_G5 TTGTTGTTATATATATTATCATTATTATATATAGTATCATTATTATATATATTGTTGTTA [180]

151_G6 TTGTTGTTATATATATTATCATTATTATATATAGTATCATTATTATATATATTGTTGTTA [180]

151_G7 TTGTTGTTATATATATTATCATTATTATATATAGTATCATTATTATATATATTGTTGTTA [180]

151_G1(3D7) TATATAGTATCATTATTAAATATATTATCATTATTAAATATATTATCATTATTAAATATA [240]

151_G2 TATATA------------------TTATCATTATTAAATATATTATCATTATTAAATATA [240]

151_G3 TATATAGTATCATTATTAAATATATTATCATTATTAAATATATTATCATTATTAAATATA [240]

151_G4 TATATAGTATCATTATTAAATATATTATCATTATTAAATATATTATCATTATTAAATATA [240]

151_G5 TATATAGTATCATTATTAAATATATTATCATTATTAAATATATTATCATTATTAAATATA [240]

151_G6 TATATAGTATCATTATTAAATATATTATCATTATTAAATATATTATCATTATTAAATATA [240]

151_G7 TATATAGTATCATTATTAAATATATTATCATTATTAAATATATTATCATTATTAAATATA [240]

151_G1(3D7) TTATCATTATTA------------------TTGAGGTTATTATTATTCATATTGTTTTCA [300]

151_G2 TTATCATTATTA------------------TTGAGGTTATTATTATTCATATTGTTTTCA [300]

151_G3 TTATCATTATTA------------------TTGAGGTTATTATTATTCATATTGTTTTCA [300]

151_G4 TTATCATTATTA------------------TTGAGGTTATTATTATTCATATTGTTTTCA [300]

151_G5 TTATCATTATTAAATATATTATCATTATTATTGAGGTTATTATTATTCATATTGTTTTCA [300]

151_G6 TTATCATTATTA------------------TTGAGGTTATTATTATTCATATTGTTTTCA [300]

151_G7 TTATCATTATTA------------------TTGAGGTTATTATTATTCATATTGTTTTCA [300]

151_G1(3D7) TTCGATTCATCCTTATTAATTACCTTATGATCATTCATACTATCATGATGAGCAACTTCA [360]

151_G2 TTCGACTCATCCTTATTAATTACCTTATGATCATTCATACTATCATGATGAGCAACTTCA [360]

151_G3 TTCGATTCATCCTTATTAATTACCTTATGATCATTCATACTATCATGATGAGCAACTTCA [360]

151_G4 TTCGATTCATCCTTATTAATTACCTTATGATCATTCATACTATCATGATGAGCAACTTCA [360]

151_G5 TTCGATTCATCCTTATTAATTACCTTATGATCATTCATACTATCATGATGAGCAACTTCA [360]

151_G6 TTCGATTCATCCTTATTAATTACCTTATGATCATTCATACTATCATGATGAGCAACTTCA [360]

151_G7 TTCGATTCATCCTTATTAATTACCTTATGATCATTCATACTATCATGATGAGCAACTTCA [360]

151_G1(3D7) TTACCTTTCATTATATT---------------TTCTTTCATTATATTATCCTTCATATTA [420]

151_G2 TTACCTTTCATTATATT---------------TTCTTTCATTATATTATCCTTCATATTA [420]

151_G3 TTACCTTTCATTATATT---------------TTCTTTCATTATATTATCCTTCATATTA [420]

151_G4 TTACCTTTCATTATATTATCTTTCATTATATTTTCTTTCATTATATTATCCTTCATATTA [420]

151_G5 TTACCTTTCATTATATT---------------TTCTTTCATTATATTATCCTTCATATTA [420]

151_G6 TTACCTTTCATTATATTATCTTTCATTATATTTTCTTTCATTATATTATCCTTCATATTA [420]

151_G7 TTACCTTTCATTATATTATCTTTCATTATATTTTCTTTCATTATATTATCCTTCATATTA [420]

151_G1(3D7) TTTTCCTTCATA---------------------------------------------ATA [480]

151_G2 TTTTCCTTCATA---------------------------------------------ATA [480]

151_G3 TTTTCCTTCATATTATTTTCCTTCATA------------------------------ATA [480]

151_G4 TTTTCCTTCATATTATTTTCCTTCATATTATTTTCCTTC---------------ATAATA [480]

151_G5 TTTTCCTTCATA---------------------------------------------ATA [480]

151_G6 TTTTCCTTCATATTATTTTCCTTCATATTATTTTCCTTCATATTATTTTCCTTCATAATA [480]

151_G7 TTTTCCTTCATATTATTTTCCTTCATA------------------------------ATA [480]

151_G1(3D7) TTATCATTACAATTATTTATGTTTGTATTTATATTTAGCACCCCCTGTTGCTGTTCACCT [540]

151_G2 TTATCATTACAATTATTTATGTTTGTATTTATATTTAGCACCCCCTGTTGCTGTTCACCT [540]

151_G3 TTATCATTACAATTATTTATGTTTGTATTTATATTTAGCACCCCCTGTTGCTGTTCACCT [540]

151_G4 TTATCATTACAATTATTTATGTTTGTATTTATATTTAGCACCCCCTGTTGCTGTTCACCT [540]

151_G5 TTATCATTACAATTATTTATGTTTGTATTTATATTTAGCACCCCCTGTTGCTGTTCACCT [540]

151_G6 TTATCATTACAATTATTTATGTTTGTATTTATATTTAGCACCCCCTGTTGCTGTTCACCT [540]

151_G7 TTATCATTACAATTATTTATGTTTGTATTTATATTTAGCACCCCCTGTTGCTGTTCACCT [540]

151_G1(3D7) TCATTTAGACAATTATTTTTATTTTCCTGTTTTTCCTCATTTTTGTTGATTCCAAATATT [600]

151_G2 TCATTTAGACAATTATTTTTATTTTCCTGTTTTTCCTCATTTTTGTTGATTCCAAATATT [600]

151_G3 TCATTTAGACAATTATTTTTATTTTCCTGTTTTTCCTCATTTTTGTTGATTCCAAATATT [600]

151_G4 TCTTTTAGACAATTATTTTTATTTTCCTGTTTTTCCTCATTTTTGTTGATTCCAAATATT [600]

151_G5 TCATTTAGACAATTATTTTTATTTTCCTGTTTTTCCTCATTTTTGTTGATTCCAAATATT [600]

151_G6 TCATTTAGACAATTATTTTTATTTTCCTGTTTTTCCTCATTTTTGTTGATTCCAAATATT [600]

151_G7 TCATTTAGACAATTATTTTTATTTTCCTGTTTTTCCTCATTTTTGTTGATTCCAAATATT [600]

151_G1(3D7) TTATCTTGTTCCTTTAATTTCTCAATATATTTTATAATTTGGACAATATATGCTTCATCC [660]

151_G2 TTATCTTGTTCCTTTAATTTCTCAATATATTTTATAATTTGGACAATATATGCTTCATCC [660]

151_G3 TTATCTTGTTCCTTTAATTTCTCAATATATTTTATAATTTGGACAATATATGCTTCATCC [660]

151_G4 TTATCTTGTTCCTTTAATTTCTCAATATATTTTATAATTTGGACAATATATGCTTCATCC [660]

151_G5 TTATCTTGTTCCTTTAATTTCTCAATATATTTTATAATTTGGACAATATATGCTTCATCC [660]

151_G6 TTATCTTGTTCCTTTAATTTCTCAATATATTTTATAATTTGGACAATATATGCTTCATCC [660]

151_G7 TTATCTTGTTCCTTTAATTTCTCAATATATTTTATAATTTGGACAATATATGCTTCATCC [660]

151_G1(3D7) TTTTCATTAGGTTCTACAGGGAAAATTTTTCTTTGTTTTTTTTGTTTTCTGTTTATAAAG [720]

151_G2 TTTTCATTAGGTTCTACAGGGAAAATTTTTCTTTGTTTTTTTTGTTTTCTGTTTATAAAG [720]

151_G3 TTTTCATTAGGTTCTACAGGGAAAATTTTTCTTTGTTTTTTTTGTTTTCTGTTTATAAAG [720]

151_G4 TTTTCATTAGGTTCTACAGGGAAAATTTTTCTTTGTTTTTTTTGTTTTCTGTTTATAAAG [720]

151_G5 TTTTCATTAGGTTCTACAGGGAAAATTTTTCTTTGTTTTTTTTGTTTTCTGTTTATAAAG [720]

151_G6 TTTTCATTAGGTTCTACAGGGAAAATTTTTCTTTGTTTTTTTTGTTTTCTGTTTATAAAG [720]

151_G7 TTTTCATTAGGTTCTACAGGGAAAATTTTTCTTTGTTTTTTTTGTTTTCTGTTTATAAAG [720]

151_G1(3D7) GTTAAAATATAACTTGACTTAAAATTATCATAGTGTATATAGCAATTTGTACATATTTCT [780]

151_G2 GTTAAAATATAACTTGACTTAAAATTATCATAGTGTATATAGCAATTTGTACATATTTCT [780]

151_G3 GTTAAAATATAACTTGACTTAAAATTATCATAGTGTATATAGCAATTTGTACATATTTCT [780]

151_G4 GTTAAAATATAACTTGACTTAAAATTATCATAGTGTATATAGCAATTTGTACATATTTCT [780]

151_G5 GTTAAAATATAACTTGACTTAAAATTATCATAGTGTATATAGCAATTTGTACATATTTCT [780]

151_G6 GTTAAAATATAACTTGACTTAAAATTATCATAGTGTATATAGCAATTTGTACATATTTCT [780]

151_G7 GTTAAAATATAACTTGACTTAAAATTATCATAGTGTATATAGCAATTTGTACATATTTCT [780]

151_G1(3D7) TTGTTCTTTTGAACGTTTTTATTTAAAGTTCTTATATCA [819]

151_G2 TTGTTCTTTTGAACGTTTTTATTTAAAGTTCTTATATCA [819]

151_G3 TTGTTCTTTTGAACGTTTTTATTTAAAGTTCTTATATCA [819]

151_G4 TTGTTCTTTTGAACGTTTTTATTTAAAGTTCTTATATCA [819]

151_G5 TTGTTCTTTTGAACGTTTTTATTTAAAGTTCTTATATCA [819]

151_G6 TTGTTCTTTTGAACGTTTTTATTTAAAGTTCTTATATCA [819]

151_G7 TTGTTCTTTTGAACGTTTTTATTTAAAGTTCTTATATCA [819]

Seven alleles were found in K13_151 438 *Plasmodium falciparum* from the five southern provinces in Laos. Allele G1 is identical to that of PF3D7. DNA Data Bank of Japan (DDBJ) accession numbers: LC432344 – LC432350

**K13_159 allele**

159_3D7 TTTATTAAATCTTCATAAACATTTTTTACAAGTAGTATGTCATTAACTATATTAGTTATG [ 60]

159_H06 TTTATTAAATCTTCATAAACATTTTTTACAAGTAGTATGTCATTAACTATATTAGTTATG [ 60]

159_H09 TTTATTAAATCTTCATAAACATTTTTTACAAGTAGTATGTCATTAACTATATTAGTTATG [ 60]

159_H23 TTTATTAAATCTTCATAAACATTTTTTACAAGTAGTATGTCATTAACTATATTAGTTATG [ 60]

159_H25 TTTATTAAATCTTCATAAACATTTTTTACAAGTAGTATGTCATTAACTATATTAGTTATG [ 60]

159_H31 TTTATTAAATCTTCATAAACATTTTTTACAAGTAGTATGTCATTAACTATATTAGTTATG [ 60]

159_H34 TTTATTAAATCTTCATAAACATTTTTTACAAGTAGTATGTCATTAACTATATTAGTTATG [ 60]

159_H35 TTTATTAAATCTTCATAAACATTTTTTACAAGTAGTATGTCATTAACTATATTAGTTATG [ 60]

159_H52 TTTATTAAATCTTCATAAACATTTTTTACAAGTAGTATGTCATTAACTATATTAGTTATG [ 60]

159_H53 TTTATTAAATCTTCATAAACATTTTTTACAAGTAGTATGTCATTAACTATATTAGTTATG [ 60]

159_H54 TTTATTAAATCTTCATAAACATTTTTTACAAGTAGTATGTCATTAACTATATTAGTTATG [ 60]

159_H58 TTTATTAAATCTTCATAAACATTTTTTACAAGTAGTATGTCATTAACTATATTAGTTATG [ 60]

159_H59 TTTATTAAATCTTCATAAACATTTTTTACAAGTAGTATGTCATTAACTATATTAGTTATG [ 60]

159_H60 TTTATTAAATCTTCATAAACATTTTTTACAAGTAGTATGTCATTAACTATATTAGTTATG [ 60]

159_H61 TTTATTAAATCTTCATAAACATTTTTTACAAGTAGTATGTCATTAACTATATTAGTTATG [ 60]

159_H64 TTTATTAAATCTTCATAAACATTTTTTACAAGTAGTATGTCATTAACTATATTAGTTATG [ 60]

159_H70 TTTATTAAATCTTCATAAACATTTTTTACAAGTAGTATGTCATTAACTATATTAGTTATG [ 60]

159_3D7 GTATCATTTTTTTGATTTATAATATTTTGAAGGTTCTTATTTTCTTCTTCATATTCTTTT [120]

159_H06 GTATCATTCTTTTGATTTATAATATTTTGAAGGTTCTTATTTTCTTCTTCATATTCTTTT [120]

159_H09 GTATCATTTTTTTGATTTATAATATTTTGAAGGTTCTTATTTTCTTCTTCATATTCTTTT [120]

159_H23 GTATCATTTTTTTGATTTATAATATTTTGAAGGTTCTTATTTTCTTCTTCATATTCTTTT [120]

159_H25 GTATCATTTTTTTGATTTATAATATTTTGAAGGTTCTTATTTTCTTCTTCATATTCTTTT [120]

159_H31 GTATCATTTTTTTGATTTATAATATTTTGAAGGTTCTTATTTTCTTCTTCATATTCTTTT [120]

159_H34 GTATCATTTTTTTGATTTATAATATTTTGAAGGTTCTTATTTTCTTCTTCATATTCTTTT [120]

159_H35 GTATCATTTTTTTGATTTATAATATTTTGAAGGTTCTTATTTTCTTCTTCATATTCTTTT [120]

159_H52 GTATCATTCTTTTGATTTATAATATTTTGAAGGTTCTTATTTTCTTCTTCATATTCTTTT [120]

159_H53 GTATCATTTTTTTGATTTATAATATTTTGAAGGTTCTTATTTTCTTCTTCATATTCTTTT [120]

159_H54 GTATCATTTTTTTGATTTATAATATTTTGAAGGTTCTTATTTTCTTCTTCATATTCTTTT [120]

159_H58 GTATCATTTTTTTGATTTATAATATTTTGAAGGTTCTTATTTTCTTCTTCATATTCTTTT [120]

159_H59 GTATCATTTTTTTGATTTATAATATTTTGAAGGTTCTTATTTTCTTCTTCATATTCTTTT [120]

159_H60 GTATCATTCTTTTGATTTATAATATTTTGAAGGTTCTTATTTTCTTCTTCATATTCTTTT [120]

159_H61 GTATCATTCTTTTGATTTATAATATTTTGAAGGTTCTTATTTTCTTCTTCATATTCTTTT [120]

159_H64 GTATCATTCTTTTGATTTATAATATTTTGAAGGTTCTTATTTTCTTCTTCATATTCTTTT [120]

159_H70 GTATCATTTTTTTGATTTATAATATTTTGAAGGTTCTTATTTTCTTCTTCATATTCTTTT [120]

159_3D7 AATGTATTTATTAAATTGTTTATTTTTTCTTTACTCTCATTCATATTATGTGCCCATACT [180]

159_H06 AATGTATTTATTAAATTGTTTATTTTTTCTTTACTCTCATTCATATTATGTGCCCATACT [180]

159_H09 AATGTATTTATTAAATTGTTTATTTTTTCTTTACTCTCATTCATATTATGTGCCCATACT [180]

159_H23 AATGTATTTATTAAATTGTTTATTTTTTCTTTACTCTCATTCATATTATGTGCCCATACT [180]

159_H25 AATGTATTTATTAAATTGTTTATTTTTTCTTTACTCTCATTCATATTATGTGCCCATACT [180]

159_H31 AATGTATTTATTAAATTGTTTATTTTTTCTTTACTCTCATTCATATTATGTGCCCATACT [180]

159_H34 AATGTATTTATTAAATTGTTTATTTTTTCTTTACTCTCATTCATATTATGTGCCCATACT [180]

159_H35 AATGTATTTATTAAATTGTTTATTTTTTCTTTACTCTCATTCATATTATGTGCCCATACT [180]

159_H52 AATGTATTTATTAAATTGTTTATTTTTTCTTTACTCTCATTCATATTATGTGCCCATACT [180]

159_H53 AATGTATTTATTAAATTGTTTATTTTTTCTTTACTCTCATTCATATTATGTGCCCATACT [180]

159_H54 AATGTATTTATTAAATTGTTTATTTTTTCTTTACTCTCATTCATATTATGTGCCCATACT [180]

159_H58 AATGTATTTATTAAATTGTTTATTTTTTCTTTACTCTCATTCATATTATGTGCCCATACT [180]

159_H59 AATGTATTTATTAAATTGTTTATTTTTTCTTTACTCTCATTCATATTATGTGCCCATACT [180]

159_H60 AATGTATTTATTAAATTGTTTATTTTTTCTTTACTCTCATTCATATTATGTGCCCATACT [180]

159_H61 AATGTATTTATTAAATTGTTTATTTTTTCTTTACTCTCATTCATATTATGTGCCCATACT [180]

159_H64 AATGTATTTATTAAATTGTTTATTTTTTCTTTACTCTCATTCATATTATGTGCCCATACT [180]

159_H70 AATGTATTTATTAAATTGTTTATTTTTTCTTTACTCTCATTCATATTATGTGCCCATACT [180]

159_3D7 TTTTCGTTGTTACAATATTTTTCCTTATCTTTTTTATAACTTTCTATAAGGTTCTTATAT [240]

159_H06 TTTTCGTTGTTACAATATTTTTCCTTATCTTTTTTATAACTTTCTATAAGGTTCTTATAT [240]

159_H09 TTTTCGTTGTTACAATATTTTTCCTTATCTTTTTTATAACTTTCTATAAGGTTCTTATAT [240]

159_H23 TTTTCGTTGTTACAATATTTTTCCTTATCTTTTTTATAACTTTCTATAAGGTTCTTATAT [240]

159_H25 TTTTCGTTGTTACAATATTTTTCCTTATCTTTTTTATAACTTTCTATAAGGTTCTTATAT [240]

159_H31 TTTTCGTTGTTACAATATTTTTCCTTATCTTTTTTATAACTTTCTATAAGGTTCTTATAT [240]

159_H34 TTTTCGTTGTTACAATATTTTTCCTTATCTTTTTTATAACTTTCTATAAGGTTCTTATAT [240]

159_H35 TTTTCGTTGTTACAATATTTTTCCTTATCTTTTTTATAACTTTCTATAAGGTTCTTATAT [240]

159_H52 TTTTCGTTGTTACAATATTTTTCCTTATCTTTTTTATAACTTTCTATAAGGTTCTTATAT [240]

159_H53 TTTTCGTTGTTACAATATTTTTCCTTATCTTTTTTATAACTTTCTATAAGGTTCTTATAT [240]

159_H54 TTTTCGTTGTTACAATATTTTTCCTTATCTTTTTTATAACTTTCTATAAGGTTCTTATAT [240]

159_H58 TTTTCGTTGTTACAATATTTTTCCTTATCTTTTTTATAACTTTCTATAAGGTTCTTATAT [240]

159_H59 TTTTCGTTGTTACAATATTTTTCCTTATCTTTTTTATAACTTTCTATAAGGTTCTTATAT [240]

159_H60 TTTTCGTTGTTACAATATTTTTCCTTATCTTTTTTATAACTTTCTATAAGGTTCTTATAT [240]

159_H61 TTTTCGTTGTTACAATATTTTTCCTTATCTTTTTTATAACTTTCTATAAGGTTCTTATAT [240]

159_H64 TTTTCGTTGTTACAATATTTTTCCTTATCTTTTTTATAACTTTCTATAAGGTTCTTATAT [240]

159_H70 TTTTCGTTGTTACAATATTTTTCCTTATCTTTTTTATAACTTTCTATAAGGTTCTTATAT [240]

159_3D7 TCATTTATTCTTTGATTAGCTTCTTGTTCTTTTTCTTTATTCATTGTAAGGATTATATCT [300]

159_H06 TCATTTATTCTTTGATTAGCTTCTTGTTCTTTTTCTTTATTCATTGTAAGGATTATATCT [300]

159_H09 TCATTTATTCTTTGATTAGCTTCTTGTTCTTTTTCTTTATTCATTGTAAGGATTATATCT [300]

159_H23 TCATTTATTCTTTGATTAGCTTCTTGTTCTTTTTCTTTATTCATTGTAAGGATTATATCT [300]

159_H25 TCATTTATTCTTTGATTAGCTTCTTGTTCTTTTTCTTTATTCATTGTAAGGATTATATCT [300]

159_H31 TCATTTATTCTTTGATTAGCTTCTTGTTCTTTTTCTTTATTCATTGTAAGGATTATATCT [300]

159_H34 TCATTTATTCTTTGATTAGCTTCTTGTTCTTTTTCTTTATTCATTGTAAGGATTATATCT [300]

159_H35 TCATTTATTCTTTGATTAGCTTCTTGTTCTTTTTCTTTATTCATTGTAAGGATTATATCT [300]

159_H52 TCATTTATTCTTTGATTAGCTTCTTGTTCTTTTTCTTTATTCATTGTAAGGATTATATCT [300]

159_H53 TCATTTATTCTTTGATTAGCTTCTTGTTCTTTTTCTTTATTCATTGTAAGGATTATATCT [300]

159_H54 TCATTTATTCTTTGATTAGCTTCTTGTTCTTTTTCTTTATTCATTGTAAGGATTATATCT [300]

159_H58 TCATTTATTCTTTGATTAGCTTCTTGTTCTTTTTCTTTATTCATTGTAAGGATTATATCT [300]

159_H59 TCATTTATTCTTTGATTAGCTTCTTGTTCTTTTTCTTTATTCATTGTAAGGATTATATCT [300]

159_H60 TCATTTATTCTTTGATTAGCTTCTTGTTCTTTTTCTTTATTCATTGTAAGGATTATATCT [300]

159_H61 TCATTTATTCTTTGATTAGCTTCTTGTTCTTTTTCTTTATTCATTGTAAGGATTATATCT [300]

159_H64 TCATTTATTCTTTGATTAGCTTCTTGTTCTTTTTCTTTATTCATTGTAAGGATTATATCT [300]

159_H70 TCATTTATTCTTTGATTAGCTTCTTGTTCTTTTTCTTTATTCATTGTAAGGATTATATCT [300]

159_3D7 TCATTTTGTTGTTCCATTTTTTTTATTATATCTTCTTTCTCATTCTCCAATCTTTTTATG [360]

159_H06 TCATTTTGTTGTTCCATTTTTTTTATTATATCTTCTTTCTCATTCTCCAATCTTTTTATG [360]

159_H09 TCATTTTGTTGTTCCATTTTTTTTATTATATCTTCTTTCTCATTCTCCAATCTTTTTATG [360]

159_H23 TCATTTTGTTGTTCCATTTTTTTTATTATATCTTCTTTCTCATTCTCCAATCTTTTTATG [360]

159_H25 TCATTTTGTTGTTCCATTTTTTTTATTATATCTTCTTTCTCATTCTCCAATCTTTTTATG [360]

159_H31 TCATTTTGTTGTTCCATTTTTTTTATTATATCTTCTTTCTCATTCTCCAATCTTTTTATG [360]

159_H34 TCATTTTGTTGTTCCATTTTTTTTATTATATCTTCTTTCTCATTCTCCAATCTTTTTATG [360]

159_H35 TCATTTTGTTGTTCCATTTTTTTTATTATATCTTCTTTCTCATTCTCCAATCTTTTTATG [360]

159_H52 TCATTTTGTTGTTCCATTTTTTTTATTATATCTTCTTTCTCATTCTCCAATCTTTTTATG [360]

159_H53 TCATTTTGTTGTTCCATTTTTTTTATTATATCTTCTTTCTCATTCTCCAATCTTTTTATG [360]

159_H54 TCATTTTGTTGTTCCATTTTTTTTATTATATCTTCTTTCTCATTCTCCAATCTTTTTATG [360]

159_H58 TCATTTTGTTGTTCCATTTTTTTTATTATATCTTCTTTCTCATTCTCCAATCTTTTTATG [360]

159_H59 TCATTTTGTTGTTCCATTTTTTTTATTATATCTTCTTTCTCATTCTCCAATCTTTTTATG [360]

159_H60 TCATTTTGTTGTTCCATTTTTTTTATTATATCTTCTTTCTCATTCTCCAATCTTTTTATG [360]

159_H61 TCATTTTGTTGTTCCATTTTTTTTATTATATCTTCTTTCTCATTCTCCAATCTTTTTATG [360]

159_H64 TCATTTTGTTGTTCCATTTTTTTTATTATATCTTCTTTCTCATTCTCCAATCTTTTTATG [360]

159_H70 TCATTTTGTTGTTCCATTTTTTTTATTATATCTTCTTTCTCATTCTCCAATCTTTTTATG [360]

159_3D7 ATATCTTCTTTCTCATTCTCCAATCTTTTTATGATATCTTCTTTCTCATTCTCCAACCTT [420]

159_H06 ATATCTTCTTTCTCATTCTCCAATCTTTTTATG--------------------------- [420]

159_H09 ATATCTTCTTTCTCATTCTCCAACCTTTTTATT--------------------------- [420]

159_H23 ATATCTTCTTTCTCATTCTCCAATCTTTTTATG--------------------------- [420]

159_H25 ATATCTTCTTTCTCATTCTCCAATCTTTTTATGATATCTTCTTTCTCATTCTCCAACCTT [420]

159_H31 ATATCTTCTTTCTCATTCTCCAATCTTTTTATG--------------------------- [420]

159_H34 ATATCTTCTTTCTCATTCTCCAATCTTTTTATG--------------------------- [420]

159_H35 ATATCTTCTTTCTCATTCTCCAATCTTTTTATGATATCTTCTTTCTCATTCTCCAACCTT [420]

159_H52 ATATCTTCTTTCTCATTCTCCAATCTTTTTATG--------------------------- [420]

159_H53 ATATCTTCTTTCTCATTCTCCAATCTTTTTATG--------------------------- [420]

159_H54 ATATCTTCTTTCTCATTCTCCAATCTTTTTATG--------------------------- [420]

159_H58 ATATCTTCTTTCTCATTCTCCAATCTTTTTATGATATCTTCTTTCTCATTCTCCAACCTT [420]

159_H59 ATATCTTCTTTCTCATTCTCCAATCTTTTTATG--------------------------- [420]

159_H60 ATATCTTCTTTCTCATTCTCCAATCTTTTTATG--------------------------- [420]

159_H61 ATATCTTCTTTCTCATTCTCCAATCTTTTTATG--------------------------- [420]

159_H64 ATATCTTCTTTCTCATTCTCCAATCTTTTTATT--------------------------- [420]

159_H70 ATATCTTCTTTCTCATTCTCCAATCTTTTTATGATATCTTCTTTCTCATTCTCCAACCTT [420]

159_3D7 TTTATTATATCTTCTTTTTCATTCTCGAACCCTTTTATGATATCTTCTTTCTCTTTTTCG [480]

159_H06 ------ATATCTTCTTTCTCATTCTCCAACCTTTTTATTATATCTTCTTTTTCATTCTCG [480]

159_H09 ------ATATCTTCTTTTTCATTCTCGAACCCTTTTATGATATCTTCTTTCTCTTTTTCG [480]

159_H23 ------ATATCTTCTTTCTCATTCTCCAACCTTTTTATTATATCTTCTTTTTCATTCTCG [480]

159_H25 TTTATTATATCTTCTTTTTCATTCTCGAACCCTTTTATGATATCTTCTTTCTCTTTTTCG [480]

159_H31 ------ATATCTTCTTTCTCATTCTCCAACCTTTTTATTATATCTTCTTTTTCATTCTCG [480]

159_H34 ------ATATCTTCTTTCTCATTCTCCAACCTTTTTATTATATCTTCTTTTTCATTCTCG [480]

159_H35 TTTATTATATCTTCTTTTTCATTCTCGAACCCTTTTATGATATCTTCTTTCTCTTTTTCG [480]

159_H52 ------ATATCTTCTTTCTCTTTTTCGAACCCTTTTATGATATCTTCTTTTTCATTCTCG [480]

159_H53 ------ATATCTTCTTTCTCATTCTCCAACCTTTTTATTATATCTTCTTTTTCATTCTCG [480]

159_H54 ------ATATCTTCTTTCTCATTCTCCAACCTTTTTATTATATCTTCTTTTTCATTCTCG [480]

159_H58 TTTATTATATCTTCTTTTTCATTCTCGAACCCTTTTATGATATCTTCTTTCTCTTTTTCG [480]

159_H59 ------ATATCTTCTTTCTCATTCTCCAACCTTTTTATTATATCTTCTTTTTCATTCTCG [480]

159_H60 ------ATATCTTCTTTCTCATTCTCCAACCTTTTTATTATATCTTCTTTTTCATTCTCG [480]

159_H61 ------ATATCTTCTTTCTCATTCTCCAACCTTTTTATTATATCTTCTTTTTCATTCTCG [480]

159_H64 ------ATATCTTCTTTTTCATTCTCGAACCCTTTTATGATATCTTCTTTCTCTTTTTCG [480]

159_H70 TTTATTATATCTTCTTTTTCATTCTCGAACCCTTTTATGATATCTTCTTTCTCTTTTTCG [480]

159_3D7 AACCCTTTTATGATATCTTCTTTCTCTTTTTCGAACCCTTTTATTACATCTTCTTTCTCT [540]

159_H06 AACCCTTTTATGATATCTTCTTTCTCTTTTTCGAACCCTTTTATGATATCTTCTTTCTCT [540]

159_H09 AACCCTTTTATGATATCTTCTTTCTCTTTTTCGAACCCTTTTATTACATCTTCTTTCTCT [540]

159_H23 AACCCTTTTATGATATCTTCTTTCTCTTTTTCGAACCCTTTTATGATATCTTCTTTCTCT [540]

159_H25 AACCCTTTTATGATATCTTCTTTCTCTTTTTCGAACCCTTTTATGATATCTTCTTTCTCT [540]

159_H31 AACCCTTTTATGATATCTTCTTTCTCTTTTTCGAACCCTTTTATGATATCTTCTTTCTCT [540]

159_H34 AACCCTTTTATGATATCTTCTTTCTCTTTTTCGAACCCTTTTATGATATCTTCTTTCTCT [540]

159_H35 AACCCTTTTATGATATCTTCTTTCTCTTTTTCGAACCCTTTTATTACATCTTCTTTCTCT [540]

159_H52 AACCCTTTTATGATATCTTCTTTCTCTTTTTCGAACCCTTTTATTACATCTTCTTTCTCT [540]

159_H53 AACCCTTTTATGATATCTTCTTTCTCTTTTTCGAACCCTTTTATGATATCTTCTTTCTCT [540]

159_H54 AACCCTTTTATGATATCTTCTTTCTCTTTTTCGAACCCTTTTATGATATCTTCTTTCTCT [540]

159_H58 AACCCTTTTATGATATCTTCTTTCTCTTTTTCGAACCCTTTTATGATATCTTCTTTCTCT [540]

159_H59 AACCCTTTTATGATATCTTCTTTCTCTTTTTCGAACCCTTTTATGATATCTTCTTTCTCT [540]

159_H60 AACCCTTTTATGATATCTTCTTTCTCTTTTTCGAACCCTTTTATGATATCTTCTTTCTCT [540]

159_H61 AACCCTTTTATGATATCTTCTTTCTCTTTTTCGAACCCTTTTATGATATCTTCTTTCTCT [540]

159_H64 AACCCTTTTATGATATCTTCTTTCTCTTTTTCGAACCCTTTTATGATATCTTCTTTCTCT [540]

159_H70 AACCCTTTTATGATATCTTCTTTCTCTTTTTCGAACCCTTTTATGATATCTTCTTTCTCT [540]

159_3D7 TTTTCGAACCCTTTTATGATATCTTCTTTCTCTTTTTCGAACCCTTTTATTACATCTTCT [600]

159_H06 TTTTCGAACCCTTTTATTACATCTTCTTTCTCTTTTTCGAACCCTTTTATTACATCTTCT [600]

159_H09 TTTTCGAACCCTTTTATTACATCTTCTTTCTCTTTTTCGAACCCTTTTATTACATCTTCT [600]

159_H23 TTTTCGAACCCTTTTATGATATCTTCTTTCTCTTTTTCGAACCCTTTTATTACATCTTCT [600]

159_H25 TTTTCGAACCCTTTTATTACATCTTCTTTCTCTTTTTCGAACCCTTTTATTACATCTTCT [600]

159_H31 TTTTCGAACCCTTTTATTACATCTTCTTTCTCTTTTTCGAACCCTTTTATTACATCTTCT [600]

159_H34 TTTTCGAACCCTTTTATTACATCTTCTTTCTCTTTTTCGAACCCTTTTATTACATCTTCT [600]

159_H35 TTTTCGAACCCTTTTATTACATCTTCTTTCTCTTTTTCGAACCCTTTTATTACATCTTCT [600]

159_H52 TTTTCGAACCCTTTTATTACATCTTCTTTCTCTTTTTCGAACCCTTTTATTACATCTTCT [600]

159_H53 TTTTCGAACCCTTTTATGATATCTTCTTTCTCTTTTTCCAACCCTTTTATTACATCTTCT [600]

159_H54 TTTTCGAACCCTTTTATGATATCTTCTTTCTCTTTTTCGAACCCTTTTATTACATCTTCT [600]

159_H58 TTTTCGAACCCTTTTATGATATCTTCTTTCTCTTTTTCGAACCCTTTTATTACATCTTCT [600]

159_H59 TTTTCGAACCCTTTTATTACATCTTCTTTCTCTTTTTCGAACCCTTTTATTACATCTTCT [600]

159_H60 TTTTCGAACCCTTTTATGATATCTTCTTTCTCTTTTTCGAACCCTTTTATTACATCTTCT [600]

159_H61 TTTTCGAACCCTTTTATGATATCTTCTTTCTCTTTTTCGAACCCTTTTATTACATCTTCT [600]

159_H64 TTTTCGAACCCTTTTATTACATCTTCTTTCTCTTTTTCGAACCCTTTTATTACATCTTCT [600]

159_H70 TTTTCGAACCCTTTTATTACATCTTCTTTCTCTTTTTCGAACCCTTTTATTACATCTTCT [600]

159_3D7 TTCTCTTTCTCCAATCTTTTTATTACATCTTCTTTCTCTTTCTCCAATCTTTTTATTACA [660]

159_H06 TTCTCTTTTTCGAACCCTTTTATTACATCTTCTTTCTCTTTCTCCAATCTTTTTATTACA [660]

159_H09 TTCTCTTTCTCCAATCTTTTTATTACATCTTCTTTCTCTTTCTCCAATCTTTTTAT---- [660]

159_H23 TTCTCTTTTTCGAACCCTTTTATTACATCTTCTTTCTCTTTCTCCAATCTTTTTATTACA [660]

159_H25 TTCTCTTTCTCCAATCTTTTTATTACATCTTCTTTCTCTTTCTCCAATCTTTTTATTACA [660]

159_H31 TTCTCTTTCTCCAATCTTTTTATTACATCTTCTTTCTCTTTCTCCAATCTTTTTATTACA [660]

159_H34 TTCTCTTTTTCGAACCCTTTTATTACATCTTCTTTCTCTTTCTCCAATCTTTTTATTACA [660]

159_H35 TTCTCTTTCTCCAATCTTTTTATTACATCTTCTTTCTCTTTCTCCAATCTTTTTATTACA [660]

159_H52 TTCTCTTTCTCCAATCTTTTTATTACATCTTCTTTCTCTTTCTCCAATCTTTTTATTACA [660]

159_H53 TTCTCTTTTTCCAACCCTTTTATTACATCTTCTTTCTCTTTCTCCAATCTTTTTAT---- [660]

159_H54 TTCTCTTTTTCGAACCCTTTTATTACATCTTCTTTCTCTTTCTCCAATCTTTTTAT---- [660]

159_H58 TTCTCTTTTTCGAACCCTTTTATTACATCTTCTTTCTCTTTCTCCAATCTTTTTATTACA [660]

159_H59 TTCTCTTTCTCCAATCTTTTTATTACATCTTCTTTCTCTTTCTCCAATCTTTTTAT---- [660]

159_H60 TTCTCTTTCTCCAATCTTTTTATTACATCTTCTTTCTCTTTCTCCAATCTTTTTATTACA [660]

159_H61 TTCTCTTTTTCGAACCCTTTTATTACATCTTCTTTCTCTTTCTCCAATCTTTTTATTACA [660]

159_H64 TTCTCTTTTTCGAACCCTTTTATTACATCTTCTTTCTCTTTCTCCAATCTTTTTATTACA [660]

159_H70 TTCTCTTTCTCCAATCTTTTTATTACATCTTCTTTCTCTTTTTCCAATCTTTTTATTACA [660]

159_3D7 TCTTCTTTCTCTTTCTCCAATCTTTTTATCACATCTTCCAGATGTTCC [708]

159_H06 TCTTCTTTCTCTTTCTCCAATCTTTTTATCACATCTTCCAGATGTTCC [708]

159_H09 -----------------------------CACATCTTCCAGATGTTCC [708]

159_H23 TCTTCTTTCTCTTTCTCCAATCTTTTTATCACATCTTCCAGATGTTCC [708]

159_H25 TCTTCTTTCTCTTTCTCCAATCTTTTTATCACATCTTCCAGATGTTCC [708]

159_H31 TCTTCTTTCTCTTTCTCCAATCTTTTTATCACATCTTCCAGATGTTCC [708]

159_H34 TCTTCTTTCTCTTTCTCCAATCTTTTTATCACATCTTCCAGATGTTCC [708]

159_H35 TCTTCTTTCTCTTTCTCCAATCTTTTTATCACATCTTCCAGATGTTCC [708]

159_H52 TCTTCTTTCTCTTTCTCCAATCTTTTTATCACATCTTCCAGATGTTCC [708]

159_H53 -----------------------------CACATCTTCCAGATGTTCC [708]

159_H54 -----------------------------CACATCTTCCAGATGTTCC [708]

159_H58 TCTTCTTTCTCTTTCTCCAATCTTTTTATCACATCTTCCAGATGTTCC [708]

159_H59 -----------------------------CACATCTTCCAGATGTTCC [708]

159_H60 TCTTCTTTCTCTTTCTCCAATCTTTTTATCACATCTTCCAGATGTTCC [708]

159_H61 TCTTCTTTCTCTTTCTCCAATCTTTTTATCACATCTTCCAGATGTTCC [708]

159_H64 TCTTCTTTCTCTTTCTCCAATCTTTTTATCACATCTTCCAGATGTTCC [708]

159_H70 TCTTCTTTCTCTTTCTCCAATCTTTTTATCACATCTTCCAGATGTTCC [708]

Sixteen alleles were found in K13_159 438 *Plasmodium falciparum* from the five southern provinces in Laos. DDBJ accession numbers: LC432351 – LC432366.
