# Supplementary material for: Heterogeneous distribution of k13 mutations in Plasmodium falciparum in Laos
Source: Malar J. 2018 Dec 27;17:483. doi: 10.1186/s12936-018-2625-6 (PMC6307170; doi:10.1186/s12936-018-2625-6)
Supplement: Supplementary file 6 — Additional file 6. Haplotypes based on the two flanking loci of the k13 gene in the six provinces, summarized by frequency of the haplotypes. [file 12936_2018_2625_MOESM6_ESM.docx]

**Additional File 6**  Haplotypes based on the two flanking loci of the *k13* gene in the six provinces,

summarized by frequency of the haplotypes

| **Provine** | **Combination of the two alleles** | **Haplotype ID** | **Genotype of the K13** | **Frequency** |
| --- | --- | --- | --- | --- |
| Savannakhet | 151_G3/159_H25 | H01 | C580Y | 63 |
| Salavan | 151_G3/159_H25 | H01 | C580Y | 54 |
| Salavan | 151_G3/159_H25 | H01 | WT | 1 |
| Sekong | 151_G3/159_H25 | H01 | C580Y | 17 |
| Sekong | 151_G3/159_H25 | H01 | Y493H | 2 |
| Sekong | 151_G3/159_H25 | H01 | WT | 1 |
| Attapeu | 151_G3/159_H25 | H01 | C580Y | 16 |
| Champasak | 151_G3/159_H25 | H01 | C580Y | 24 |
| Phongsaly | 151_G3/159_H25 | H01 | C580Y | 3 |
| Salavan | 151_G1/159_H09 | H02 | WT | 5 |
| Salavan | 151_G1/159_H09 | H02 | C580Y | 1 |
| Attapeu | 151_G1/159_H09 | H02 | C580Y | 29 |
| Champasak | 151_G1/159_H09 | H02 | C580Y | 12 |
| Sekong | 151_G2/159_H52 | H03 | Y493H | 32 |
| Sekong | 151_G2/159_H52 | H03 | WT | 1 |
| Attapeu | 151_G2/159_H52 | H03 | Y493H | 1 |
| Attapeu | 151_G2/159_H06 | H04 | C580Y | 8 |
| Attapeu | 151_G2/159_H06 | H04 | R539T | 2 |
| Attapeu | 151_G2/159_H06 | H04 | WT | 4 |
| Champasak | 151_G2/159_H06 | H04 | C580Y | 5 |
| Champasak | 151_G2/159_H06 | H04 | R539T | 6 |
| Champasak | 151_G2/159_H06 | H04 | C580Y/R539T | 1 |
| Sekong | 151_G7/159_H64 | H05 | WT | 19 |
| Sekong | 151_G7/159_H64 | H05 | Y493H | 1 |
| Savannakhet | 151_G1/159_H34 | H06 | WT | 4 |
| Salavan | 151_G1/159_H34 | H06 | WT | 8 |
| Sekong | 151_G1/159_H34 | H06 | WT | 4 |
| Savannakhet | 151_G4/159_H34 | H07 | WT | 4 |
| Attapeu | 151_G4/159_H34 | H07 | C580Y | 1 |
| Champasak | 151_G4/159_H34 | H07 | R539T | 6 |
| Champasak | 151_G4/159_H34 | H07 | WT | 4 |
| Savannakhet | 151_G1/159_H25 | H08 | C580Y | 4 |
| Salavan | 151_G1/159_H25 | H08 | WT | 1 |
| Attapeu | 151_G1/159_H25 | H08 | C580Y | 7 |
| Champasak | 151_G1/159_H25 | H08 | C580Y | 2 |
| Attapeu | 151_G4/159_H54 | H09 | C580Y | 1 |
| Attapeu | 151_G4/159_H54 | H09 | R539T | 4 |
| Champasak | 151_G4/159_H54 | H09 | C580Y | 1 |
| Champasak | 151_G4/159_H54 | H09 | WT | 1 |
| Champasak | 151_G4/159_H54 | H09 | C580Y/R539T | 1 |
| Champasak | 151_G4/159_H54 | H09 | R539T | 6 |
| Sekong | 151_G1/159_H06 | H10 | WT | 3 |
| Attapeu | 151_G1/159_H06 | H10 | C580Y | 2 |
| Champasak | 151_G1/159_H06 | H10 | C580Y | 1 |
| Champasak | 151_G1/159_H06 | H10 | WT | 1 |
| Savannakhet | 151_G3/159_H34 | H11 | WT | 4 |
| Champasak | 151_G3/159_H34 | H11 | C580Y | 1 |
| Champasak | 151_G3/159_H34 | H11 | WT | 1 |
| Salavan | 151_G2/159_H23 | H12 | WT | 5 |
| Attapeu | 151_G4/159_H06 | H13 | WT | 1 |
| Champasak | 151_G4/159_H06 | H13 | C580Y | 3 |
| Champasak | 151_G4/159_H06 | H13 | R539T | 1 |
| Sekong | 151_G1/159_H23 | H14 | WT | 4 |
| Sekong | 151_G1/159_H58 | H15 | WT | 3 |
| Attapeu | 151_G1/159_H58 | H15 | WT | 1 |
| Savannakhet | 151_G1/159_H61 | H16 | WT | 3 |
| Salavan | 151_G1/159_H61 | H16 | WT | 1 |
| Attapeu | 151_G2/159_H25 | H17 | C580Y | 2 |
| Champasak | 151_G2/159_H25 | H17 | C580Y | 2 |
| Salavan | 151_G4/159_H25 | H18 | WT | 1 |
| Attapeu | 151_G4/159_H25 | H18 | C580Y/R539T | 1 |
| Attapeu | 151_G4/159_H25 | H18 | R539T | 1 |
| Champasak | 151_G4/159_H25 | H18 | C580Y | 1 |
| Attapeu | 151_G1/159_H54 | H19 | C580Y | 1 |
| Champasak | 151_G1/159_H54 | H19 | C580Y | 2 |
| Savannakhet | 151_G2/159_H34 | H20 | WT | 1 |
| Champasak | 151_G2/159_H34 | H20 | WT | 2 |
| Salavan | 151_G4/159_H23 | H21 | WT | 2 |
| Sekong | 151_G4/159_H23 | H21 | WT | 1 |
| Salavan | 151_G4/159_H31 | H22 | WT | 2 |
| Salavan | 151_G4/159_H31 | H22 | C580Y | 1 |
| Salavan | 151_G1/159_H35 | H23 | WT | 1 |
| Attapeu | 151_G1/159_H35 | H23 | WT | 1 |
| Savannakhet | 151_G3/159_H23 | H24 | WT | 2 |
| Savannakhet | 151_G6/159_H34 | H25 | WT | 2 |
| Savannakhet | 151_G7/159_H60 | H26 | WT | 2 |
| Savannakhet | 151_G1/159_H31 | H27 | WT | 1 |
| Champasak | 151_G1/159_H59 | H28 | C580Y | 1 |
| Champasak | 151_G2/159_H54 | H29 | C580Y | 1 |
| Champasak | 151_G3/159_H70 | H30 | C580Y/R539T | 1 |
| Attapeu | 151_G4/159_H09 | H31 | WT | 1 |
| Salavan | 151_G4/159_H35 | H32 | WT | 1 |
| Attapeu | 151_G4/159_H53 | H33 | C580Y | 1 |
| Champasak | 151_G4/159_H58 | H34 | WT | 1 |
| Salavan | 151_G4/159_H59 | H35 | WT | 1 |
| Attapeu | 151_G5/159_H15 | H36 | WT | 1 |
| Salavan | 151_G6/159_H09 | H37 | WT | 1 |
| Total |  |  |  | 441 |

WT: Wild type
