# Supplementary material for: Heterogeneous distribution of k13 mutations in Plasmodium falciparum in Laos
Source: Malar J. 2018 Dec 27;17:483. doi: 10.1186/s12936-018-2625-6 (PMC6307170; doi:10.1186/s12936-018-2625-6)
Supplement: Supplementary file 7 — Additional file 7. Bivariate analyses to identify an association between variables and Plasmodium falciparum infections with the k13 mutations in the five provinces. [file 12936_2018_2625_MOESM7_ESM.docx]

**Additional File 7**  Bivariate analyses to identify an association between variables and *Plasmodium falciparum* infections with the *k13* mutations in the five provinces

| **Status** | | | **Total** | **No. *k13* mutation** | **(%)** | **Chi-test** | **Note** |
| --- | --- | --- | --- | --- | --- | --- | --- |
| Total |  |  | 1,151 | 639 | 55.5 | - |  |
|  |  |  |  |  |  |  |  |
| Province | Savannakhet |  | 254 | 71 | 28.0 | < 0.001 |  |
|  | Salavan |  | 216 | 126 | 58.3 |  |  |
|  | Sekong |  | 140 | 54 | 38.6 |  |  |
|  | Attapeu |  | 123 | 85 | 69.1 |  |  |
|  | Champasak |  | 418 | 303 | 72.5 |  |  |
|  |  |  |  |  |  |  |  |
| District |  |  |  |  |  | < 0.001 | All districts |
|  | Savannakhet | Total | 254 | 71 | 28.0 | < 0.001 | Savannakhet |
|  |  | Nong | 120 | 3 | 2.5 |  |  |
|  |  | Phin | 37 | 8 | 21.6 |  |  |
|  |  | Sepon | 36 | 3 | 8.3 |  |  |
|  |  | Thapangthong | 58 | 56 | 96.6 |  |  |
|  |  | Vilabouly | 3 | 1 | 33.3 |  |  |
|  |  |  |  |  |  |  |  |
|  | Salavan | Total | 216 | 126 | 58.3 | < 0.001 | Salavan |
|  |  | Taoy | 58 | 9 | 15.5 |  |  |
|  |  | Toumlan | 88 | 53 | 60.2 |  |  |
|  |  | Vapy | 70 | 64 | 91.4 |  |  |
|  |  |  |  |  |  |  |  |
|  | Sekong | Total | 140 | 54 | 38.6 | 0.931 | Sekong |
|  |  | Lamam | 28 | 11 | 39.3 |  |  |
|  |  | Thateng | 112 | 43 | 38.4 |  |  |
|  |  |  |  |  |  |  |  |
|  | Attapeu | Total | 123 | 85 | 69.1 | 0.020 | Attapeu |
|  |  | Phouvong | 29 | 15 | 51.7 |  |  |
|  |  | Sanamxay | 91 | 69 | 75.8 |  |  |
|  |  | Saysetha | 3 | 1 | 33.3 |  |  |
|  |  |  |  |  |  |  |  |
|  | Champasak | Total | 418 | 303 | 72.5 | 0.650 | Champasak |
|  |  | Khong | 66 | 46 | 69.7 |  |  |
|  |  | Mounlapamok | 14 | 9 | 64.3 |  |  |
|  |  | Pathoumphone | 338 | 248 | 73.4 |  |  |
|  |  |  |  |  |  |  |  |
| Year | Total |  | 1,151 | 639 | 55.5 | 0.592 |  |
|  | Year 2015 |  | 559 | 307 | 54.9 |  |  |
|  | Year 2016 |  | 592 | 332 | 56.1 |  |  |
|  |  |  |  |  |  |  |  |
| Age | Median = 25.4, SD = 12.8, r = 0.187, p = 0.000 | | | | | |  |
|  |  |  |  |  |  |  |  |
| Gender | Total |  | 1,151 | 639 | 55.5 | < 0.001 |  |
|  | Male |  | 961 | 587 | 61.1 |  |  |
|  | Female |  | 190 | 52 | 27.4 |  |  |
|  |  |  |  |  |  |  |  |
|  |  |  |  |  |  |  |  |
| Occupation | Total |  | 1,151 | 639 | 55.5 | < 0.001 |  |
|  | Child |  | 104 | 17 | 16.3 |  |  |
|  | Adult |  | 1,047 | 622 | 59.4 |  |  |
|  |  |  |  |  |  |  |  |
|  | Total |  | 1,151 | 639 | 55.5 | < 0.001 |  |
|  | Agriculture |  | 847 | 527 | 62.2 |  |  |
|  | Child |  | 104 | 17 | 16.3 |  |  |
|  | Civil servant |  | 15 | 8 | 53.3 |  |  |
|  | Employee |  | 1 | 0 | 0.0 |  |  |
|  | Housewife |  | 11 | 3 | 27.3 |  |  |
|  | Military |  | 6 | 3 | 50.0 |  |  |
|  | Soldier |  | 4 | 4 | 100.0 |  |  |
|  | Student |  | 130 | 57 | 43.8 |  |  |
|  | Teacher |  | 7 | 3 | 42.9 |  |  |
|  | Vender |  | 5 | 2 | 40.0 |  |  |
|  | Village Health Volunteer |  | 1 | 0 | 0.0 |  |  |
|  | Village Leader |  | 1 | 1 | 100.0 |  |  |
|  | Worker |  | 19 | 14 | 73.7 |  |  |
|  |  |  |  |  |  |  |  |
|  | Total |  | 1,047 | 622 | 59.4 | 0.001 | Excluded Children |
|  | Agriculture |  | 847 | 527 | 62.2 |  |  |
|  | Civil servant |  | 15 | 8 | 53.3 |  |  |
|  | Employee |  | 1 | 0 | 0.0 |  |  |
|  | Housewife |  | 11 | 3 | 27.3 |  |  |
|  | Military |  | 6 | 3 | 50.0 |  |  |
|  | Soldier |  | 4 | 4 | 100.0 |  |  |
|  | Student |  | 130 | 57 | 43.8 |  |  |
|  | Teacher |  | 7 | 3 | 42.9 |  |  |
|  | Vender |  | 5 | 2 | 40.0 |  |  |
|  | Village Health Volunteer |  | 1 | 0 | 0.0 |  |  |
|  | Village Leader |  | 1 | 1 | 100.0 |  |  |
|  | Worker |  | 19 | 14 | 73.7 |  |  |
|  |  |  |  |  |  |  |  |
| Education | Total |  | 1,151 | 639 | 55.5 | < 0.001 |  |
|  | No grade completed |  | 488 | 203 | 41.6 |  |  |
|  | Primary Grade |  | 418 | 274 | 65.6 |  |  |
|  | Secondary Grade |  | 170 | 108 | 63.5 |  |  |
|  | High school |  | 58 | 46 | 79.3 |  |  |
|  | College |  | 16 | 8 | 50.0 |  |  |
|  | Higher |  | 1 | 0 | 0.0 |  |  |
|  |  |  |  |  |  |  |  |
| Marital status | Total |  | 1,151 | 639 | 55.5 | 0.002 |  |
|  | Not married/single | | 512 | 253 | 49.4 |  |  |
|  | Married |  | 625 | 377 | 60.3 |  |  |
|  | Divorced |  | 9 | 5 | 55.6 |  |  |
|  | Widowed |  | 5 | 4 | 80.0 |  |  |
|  |  |  |  |  |  |  |  |
| Religion | Total |  | 1,150 | 639 | 55.6 | < 0.001 |  |
|  | Buddhism |  | 703 | 491 | 69.8 |  |  |
|  | Christianity |  | 7 | 4 | 57.1 |  |  |
|  | Animist |  | 425 | 142 | 33.4 |  |  |
|  | Other |  | 15 | 2 | 13.3 |  |  |
|  |  |  |  |  |  |  |  |
| Ethnicity | Total |  | 1,151 | 639 | 55.5 | < 0.001 |  |
|  | Lao |  | 644 | 464 | 72.0 |  |  |
|  | Lao Theung |  | 482 | 166 | 34.4 |  |  |
|  | Lao Soung |  | 20 | 5 | 25.0 |  |  |
|  | Other |  | 5 | 4 | 80.0 |  |  |
|  |  |  |  |  |  |  |  |
| Body Temperature | Average = 38.1, SD =0 .95 r = -0.05, p = 0.128 | | | | | |  |
|  |  |  |  |  |  |  |  |
| Symptoms | Total |  | 1,151 | 639 | 55.5 | 0.264 |  |
|  | No sign & symptoms | | 1 | 0 | 0.0 |  |  |
|  | Sign & symptoms | | 1,150 | 639 | 55.6 |  |  |
|  |  |  |  |  |  |  |  |
|  | Total |  | 1,151 | 639 | 55.5 | 0.439 |  |
|  | No arthralgia |  | 1,148 | 638 | 55.6 |  |  |
|  | Arthralgia |  | 3 | 1 | 33.3 |  |  |
|  |  |  |  |  |  |  |  |
|  | Total |  | 1,151 | 639 | 55.5 | 0.432 |  |
|  | No fatigue |  | 1,147 | 636 | 55.4 |  |  |
|  | Fatigue |  | 4 | 3 | 75.0 |  |  |
|  |  |  |  |  |  |  |  |
|  | Total |  | 1,151 | 639 | 55.5 | 0.899 |  |
|  | No headache |  | 913 | 506 | 55.4 |  |  |
|  | Headache |  | 238 | 133 | 55.9 |  |  |
|  |  |  |  |  |  |  |  |
|  | Total |  | 1,151 | 639 | 55.5 | 0.264 |  |
|  | No Lethargy |  | 1,150 | 639 | 55.6 |  |  |
|  | Lethargy |  | 1 | 0 | 0.0 |  |  |
|  |  |  |  |  |  |  |  |
|  | Total |  | 1,151 | 639 | 55.5 | 0.371 |  |
|  | No lumbar pain |  | 1,150 | 638 | 55.5 |  |  |
|  | Lumbar pain |  | 1 | 1 | 100.0 |  |  |
|  |  |  |  |  |  |  |  |
|  | Total |  | 1,151 | 639 | 55.5 | 0.027 |  |
|  | No myalgia |  | 1,031 | 561 | 54.4 |  |  |
|  | Myalgia |  | 120 | 78 | 65.0 |  |  |
|  |  |  |  |  |  |  |  |
|  | Total |  | 1,151 | 639 | 55.5 | 0.371 |  |
|  | No sore throat |  | 1,150 | 638 | 55.5 |  |  |
|  | Sore throat |  | 1 | 1 | 100.0 |  |  |
|  |  |  |  |  |  |  |  |
|  | Total |  | 1,151 | 639 | 55.5 | 0.875 |  |
|  | No vertigo |  | 1,149 | 638 | 55.5 |  |  |
|  | Vertigo |  | 2 | 1 | 50.0 |  |  |
|  |  |  |  |  |  |  |  |
|  | Total |  | 1,151 | 639 | 55.5 | 0.875 |  |
|  | No headache & fatigue | | 1,149 | 638 | 55.5 |  |  |
|  | Headache & fatigue | | 2 | 1 | 50.0 |  |  |
|  |  |  |  |  |  |  |  |
|  | Total |  | 1,150 | 638 | 55.5 | 0.167 |  |
|  | No headache & myalgia | | 1,079 | 593 | 55.0 |  |  |
|  | Headache & myalgia | | 71 | 45 | 63.4 |  |  |
|  |  |  |  |  |  |  |  |
|  | Total |  | 1,151 | 639 | 55.5 | 0.371 |  |
|  | No headache & vertigo | | 1,150 | 638 | 55.5 |  |  |
|  | Headache & vertigo | | 1 | 1 | 100.0 |  |  |
|  |  |  |  |  |  |  |  |
|  | Total |  | 1,151 | 639 | 55.5 | 0.170 |  |
|  | No fever |  | 26 | 11 | 42.3 |  |  |
|  | Fever |  | 1,125 | 628 | 55.8 |  |  |
|  |  |  |  |  |  |  |  |
|  | Total |  | 1,151 | 639 | 55.5 | 0.073 |  |
|  | No thrill |  | 281 | 169 | 60.1 |  |  |
|  | Thrill |  | 870 | 470 | 54.0 |  |  |
|  |  |  |  |  |  |  |  |
|  | Total |  | 1,151 | 639 | 55.5 | < 0.001 |  |
|  | No nausea |  | 876 | 523 | 59.7 |  |  |
|  | Nausea |  | 275 | 116 | 42.2 |  |  |
|  |  |  |  |  |  |  |  |
|  | Total |  | 1,151 | 639 | 55.5 | 0.069 |  |
|  | No stomachache | | 1,098 | 616 | 56.1 |  |  |
|  | Stomachache |  | 53 | 23 | 43.4 |  |  |
|  |  |  |  |  |  |  |  |
|  | Total |  | 1,151 | 639 | 55.5 | 0.844 |  |
|  | No diarrhea |  | 1,139 | 632 | 55.5 |  |  |
|  | Diarrhea |  | 12 | 7 | 58.3 |  |  |
|  |  |  |  |  |  |  |  |
|  | Total |  | 1,151 | 639 | 55.5 | 0.012 |  |
|  | No seizure |  | 1,146 | 639 | 55.8 |  |  |
|  | Seizure |  | 5 | 0 | 0.0 |  |  |
|  |  |  |  |  |  |  |  |
|  | Total |  | 1,151 | 636 | 55.3 | < 0.001 |  |
|  | No pallor |  | 960 | 576 | 60.0 |  |  |
|  | Pallor |  | 191 | 60 | 31.4 |  |  |
|  |  |  |  |  |  |  |  |
| Start symptom | Total |  | 1151 | 639 | 55.5 | < 0.001 |  |
|  | No signs and symptoms | | 22 | 6 | 27.3 |  |  |
|  | Today |  | 66 | 44 | 66.7 |  |  |
|  | Yesterday |  | 216 | 110 | 50.9 |  |  |
|  | 2 days before |  | 306 | 183 | 59.8 |  |  |
|  | 3 days before |  | 371 | 220 | 59.3 |  |  |
|  | More than 3 days | | 170 | 76 | 44.7 |  |  |
|  |  |  |  |  |  |  |  |
| Malaria history | Total |  | 1,151 | 639 | 55.5 | < 0.001 |  |
|  | First time |  | 496 | 216 | 43.5 |  |  |
|  | 1 time |  | 177 | 96 | 54.2 |  |  |
|  | 2 times |  | 216 | 138 | 63.9 |  |  |
|  | 3 times |  | 171 | 120 | 70.2 |  |  |
|  | 4 times |  | 57 | 44 | 77.2 |  |  |
|  | 5 times |  | 23 | 17 | 73.9 |  |  |
|  | More than 6 times | | 11 | 8 | 72.7 |  |  |
|  |  |  |  |  |  |  |  |
| Bet-net | Total |  | 1,151 | 639 | 55.5 | 0.108 |  |
|  | No |  | 308 | 157 | 51.0 |  |  |
|  | Yes |  | 552 | 309 | 56.0 |  |  |
|  | Depend |  | 291 | 173 | 59.5 |  |  |

OR, odds ratio; CI, confidence interval; AOR, adjusted odds ratio
