# Supplementary material for: Heterogeneous distribution of k13 mutations in Plasmodium falciparum in Laos
Source: Malar J. 2018 Dec 27;17:483. doi: 10.1186/s12936-018-2625-6 (PMC6307170; doi:10.1186/s12936-018-2625-6)
Supplement: Supplementary file 8 — Additional file 8. PCR genotyping based on msp1 and msp2. [file 12936_2018_2625_MOESM8_ESM.docx]

**Additional File 8** PCR genotyping based on *msp1* and *msp2*

| Sample ID | Province | District | *k13* mutations | *msp1* | *msp2* |
| --- | --- | --- | --- | --- | --- |
| 490 | Sekong | Thateng | C580Y, Y493H | MAD20 | FC27 |
| 1588 | Attapeu | Sanamxay | C580Y, R539T | MAD20 | FC27, IC |
| 1651 | Champasak | Pathoumphone | C580Y, R539T | MAD20, RO33 | FC27, IC |
| 1670 | Champasak | Pathoumphone | C580Y, R539T | MAD20, RO33 | FC27, IC |
| 1829 | Champasak | Pathoumphone | C580Y, R539T | MAD20 | FC27, IC |
| 1889 | Champasak | Khong | C580Y, R539T | MAD20 | FC27 |
| 2240 | Champasak | Pathoumphone | C580Y, R539T | MAD20, RO33 | FC27 |

MAD20 and RO33 represent allele types in *msp1* gene. FC27 and IC represent allele types in *msp2* gene. Two alleles in one gene represent multi clonal infection.
