# Supplementary material for: Heterogeneous distribution of k13 mutations in Plasmodium falciparum in Laos
Source: Malar J. 2018 Dec 27;17:483. doi: 10.1186/s12936-018-2625-6 (PMC6307170; doi:10.1186/s12936-018-2625-6)
Supplement: Supplementary file 9 — Additional file 9. Frequencies of the k13 mutations in the districts. [file 12936_2018_2625_MOESM9_ESM.pptx]

## Slide 1
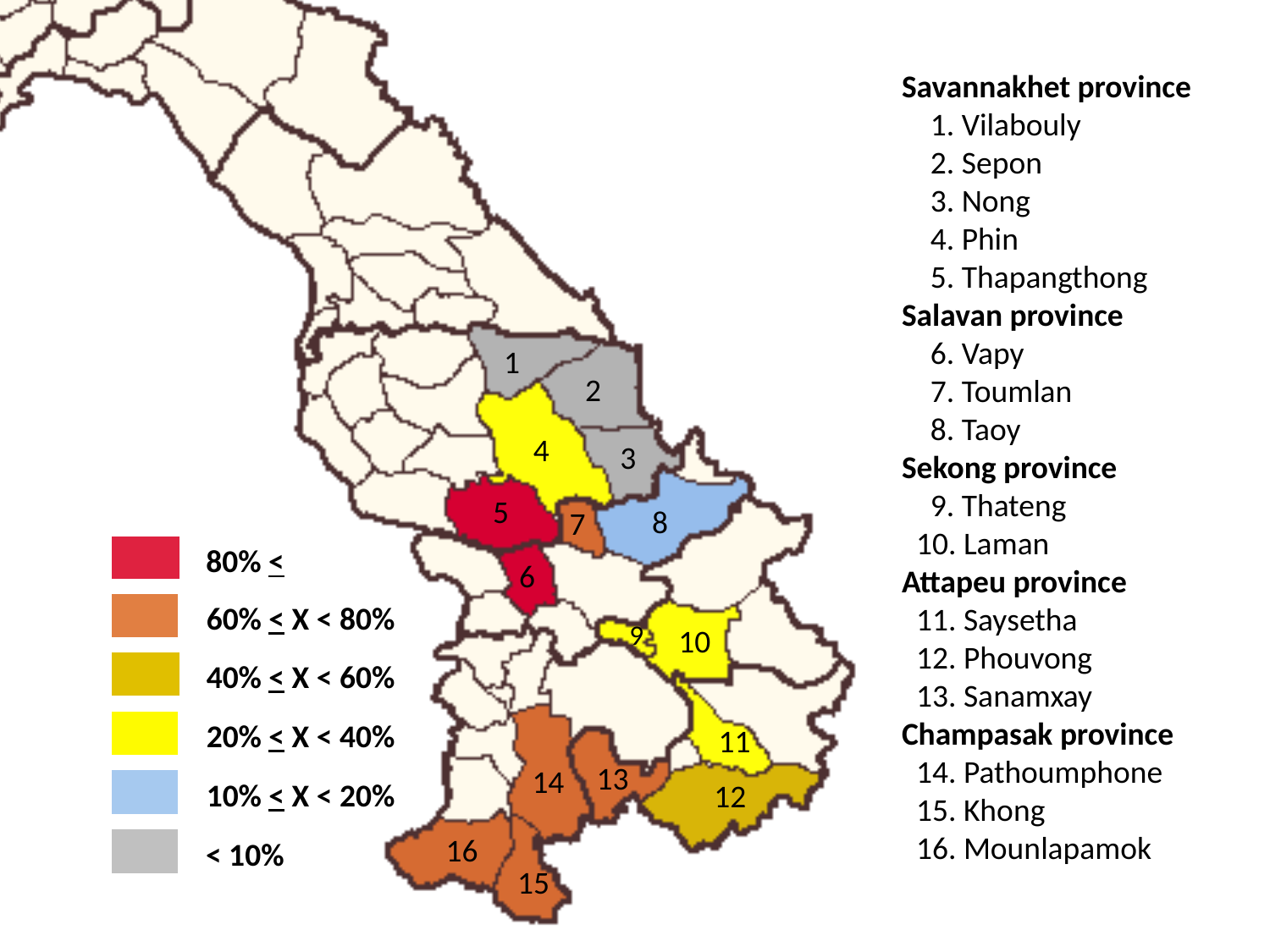

Savannakhet province
 1. Vilabouly
 2. Sepon
 3. Nong
 4. Phin
 5. Thapangthong
Salavan province
 6. Vapy
 7. Toumlan
 8. Taoy
Sekong province
 9. Thateng
 10. Laman
Attapeu province
 11. Saysetha
 12. Phouvong
 13. Sanamxay
Champasak province
 14. Pathoumphone
 15. Khong
 16. Mounlapamok
1
2
4
3
5
8
7
80% <
60% < X < 80%
40% < X < 60%
20% < X < 40%
10% < X < 20%
< 10%
6
9
10
11
13
14
12
16
15
